# A Hybrid Jiles–Atherton–Sablik Physics-Informed Convolutional Recurrent Residual Network for Magneto-Mechanical Hysteresis Prediction in Non-Oriented Electrical Steel Fe3%Si

**DOI:** 10.3390/ma19214641

**Published:** 2026-10-31

**Authors:** Malika Ikhlef, Nadia Akkari, Tarek Berghout, Kamel Srairi, Jun-Jiat Tiang, Wei Hong Lim

**Affiliations:** 1 Department of Electrical Engineering, Faculty of Technology, University of Batna 2—Mostefa Ben Boulaïd, Batna 05000, Algeria; m.ikhlef@univ-batna2.dz; 2 Laboratory of Energy Systems Modeling (LMSE), Electrical Engineering Department, Faculty of Sciences and Technology, University of Biskra, Biskra 07000, Algeria; k.srairi@univ-biskra.dz; 3 Laboratory of Electromagnetic Propulsion–Induction Systems (LSP-IE), Department of Electrical Engineering, Faculty of Technology, University of Batna 2—Mostefa Ben Boulaïd, Batna 05000, Algeria; n.akkari@univ-batna2.dz; 4 Laboratory of Automation and Manufacturing, Department of Industrial Engineering, Faculty of Technology, University of Batna 2—Mostefa Ben Boulaïd, Batna 05000, Algeria; 5 Centre for Wireless Technology, CoE for Intelligent Network, Faculty of Artificial Intelligence & Engineering, Multimedia University, Persiaran Multimedia, Cyberjaya 63100, Selangor, Malaysia; 6 Faculty of Engineering, Technology and Built Environment, UCSI University, Cheras, Kuala Lumpur 56000, Malaysia; limwh@ucsiuniversity.edu.my

**Keywords:** magnetic hysteresis, Jiles-Atherton, Sablik, deep learning, non-oriented electrical steel, magneto-mechanical coupling, eddy current

## Abstract

Accurate prediction of magnetic hysteresis under coupled mechanical and electromagnetic excitations remains a challenging problem in electrical steel modelling. Conventional phenomenological models, although physically interpretable, often suffer from limited adaptability when several effects such as stress dependence, frequency variation and non-linear domain mechanisms occur simultaneously. This work proposes a hybrid modelling framework combining a Jiles–Atherton–Sablik physical model with a deep residual correction based on a convolutional recurrent residual neural network (CRRN). The physical model provides the main magnetization mechanism, while the neural network learns the remaining discrepancy between measured and physics-based predictions. The experimental database comprises non-oriented Fe–3%Si electrical steel sheets tested under three orientations, nine stress levels (tensile/compression), three excitation frequencies, and multiple peak induction levels, totaling over 2000 BH loop files. The implemented framework optimizes the four Jiles–Atherton (JA) parameters (*k*, *c*, *a*, *α*) while keeping Sablik physical parameters (*Msat*, *λ_s_*, *σ*_0_) consistent with material properties. The optimized parameters demonstrate the model’s ability to adapt to the specific material behavior while preserving physical interpretability. The hybrid model achieves an average improvement of approximately 74% across all metrics compared to the standalone JA–Sablik model. The proposed approach improves the prediction capability of the physical model by learning unmodelled nonlinear effects while maintaining physical consistency, making it suitable for accurate hysteresis modelling under complex loading conditions.

## 1. Introduction

Electrical steel sheets are key functional materials used in electrical machines, transformers and other electromagnetic devices because their magnetic characteristics directly determine energy efficiency, power losses, thermal behaviour and operational reliability. However, the prediction of their magnetic response remains challenging because magnetization is governed by nonlinear hysteresis, rate-dependent effects, anisotropy and magneto-mechanical coupling [1,2]. In practical devices, manufacturing processes, assembly operations and operating conditions introduce mechanical stresses that modify domain wall motion and domain rotation through magnetoelastic interactions, leading to significant variations in BH loops and magnetic losses [3,4].

Among hysteresis models, JA formulation has received considerable attention because it links macroscopic hysteresis behaviour with microscopic domain mechanisms [1]. Unlike purely mathematical hysteresis operators, JA model provides physically meaningful parameters associated with irreversible domain wall pinning, reversible wall motion and anhysteretic magnetization. This physical interpretation makes it attractive for electromagnetic simulations and material characterization. Nevertheless, accurate identification of JA parameters remains difficult, especially when stress dependence, dynamic excitation and material anisotropy are considered simultaneously. The original JA model does not directly represent all mechanisms involved in stressed electrical steels, motivating several extensions including magneto-mechanical formulations based on Sablik theory, where mechanical stress is introduced through magnetoelastic coupling effects [2,5].

The increasing complexity of operating conditions has encouraged the development of hybrid physics-based and artificial intelligence approaches. Instead of replacing physical knowledge with black-box learning, hybrid approaches use physical models as structured baselines and employ neural networks to learn the remaining modelling error [6,7,8,9,10]. This strategy combines the interpretability of physical formulations with the nonlinear approximation capability of deep learning models, improving prediction accuracy while reducing the dependence on extremely large experimental datasets. Such approaches are particularly attractive for magnetic hysteresis modelling, where the dominant physical mechanisms are known but difficult to describe completely using analytical equations alone.

### 1.1. Analysis of Recent Approaches

Recent studies have explored several directions for improving hysteresis modelling accuracy. J-A based approaches remain attractive because of their computational efficiency and suitability for integration into finite-element electromagnetic simulations. However, improvements are required to reproduce minor loops, dynamic losses and stress-dependent effects. The influence of mechanical stress has been investigated through magneto-mechanical extensions of JA models [11,12]. Sablik and Jiles-Atherton introduced stress effects through an additional effective magnetic field related to magnetostriction, providing a physical explanation of the inverse magnetostrictive effect [13]. More recent models have incorporated anisotropy and stress dependent behaviour to better reproduce experimental electrical steel characteristics. Parallel developments have focused on machine-learning approaches. Neural networks have demonstrated strong capability for reproducing hysteresis trajectories under complex excitation waveforms [6,7,8,9,10]. However, purely data-driven models generally lack physical interpretation and may require extensive datasets covering all possible operating conditions [5,12].

Hybrid physics-data-driven models have therefore emerged as an important research direction because they can use a physical formulation as a structured prior while assigning the neural component only the part of the response that is difficult to reproduce analytically.

Li et al. [7] proposed a hybrid dynamic hysteresis model combining dynamic JA and Preisach descriptions through a backpropagation neural network. The JA component mainly represents domain-wall motion, whereas the Preisach component complements the description of domain rotation; the formulation also incorporates eddy-current effects. The authors validated the model experimentally on grain-oriented SiFe, non-oriented SiFe and amorphous materials under sinusoidal and distorted magnetic excitations, demonstrating that the hybrid formulation can reproduce dynamic hysteresis loops and core losses over different excitations. The approach is important because it couples complementary physical descriptions rather than relying on a purely black-box predictor. Nevertheless, mechanical stress and magneto-mechanical coupling were not included, and the neural network was used to couple physical hysteresis models rather than to learn a residual correction to a stress-aware JA-Sablik baseline.

Ni et al. [8] addressed the interpretability limitation of conventional neural hysteresis models by reformulating the Jiles–Atherton equations as a structured Jiles–Atherton Neural Network (JANN) and coupling it with a Nonlinear Autoregressive Network with eXogenous inputs (NARX). In their architecture, the weights of the JANN retain a direct correspondence with the physical parameters of the JA model, while the NARX component captures dynamic memory and rate-dependent nonlinearities. This combination enabled the resulting JA-NARX model to represent static, asymmetric and frequency-dependent hysteresis while maintaining a clearer physical interpretation than conventional black-box neural networks. The model was experimentally evaluated on a piezoelectric positioning system over excitation frequencies of 3, 15, 30 and 60 Hz. At 60 Hz, the reported peak-to-valley prediction error was 0.8190 μm, corresponding to approximately 0.82% of the full-scale range, while the average error remained close to zero. In a direct comparison, the JA-NARX model achieved lower peak-to-valley, average and standard-deviation errors than the conventional Bouc-Wen and JA models, with a reported computation time of 18 s compared with 432 s and 587 s, respectively. Nevertheless, the study was developed for voltage-displacement hysteresis in piezoelectric actuators rather than magnetic B-H hysteresis in electrical steels. Consequently, mechanical stress, magnetostriction, magnetic anisotropy and orientation-dependent behaviour were not incorporated. Moreover, the authors noted that the selected number of NARX neurons and delay steps was not fully optimized, which produced some variation in prediction accuracy with excitation frequency.

Hong et al. [9] pursued a different physics-data integration strategy by developing a physics-informed neural network for predicting magnetostriction in grain-oriented electrical steel. Their method first derived a physical magnetostriction formulation from the Jiles–Atherton model by introducing an elastic potential-energy contribution and then used a Physics-Informed Neural Network (PINN) to learn the residual associated with the governing physical equation. Unlike conventional neural networks trained only from input-output data, the PINN incorporated both prediction loss and a physics-based PDE loss, thereby constraining the learned response according to the underlying magneto-mechanical relationships. Experimental measurements of magnetic field, induction and magnetostriction were obtained under different excitation voltages and tensile stresses. A typical network consisted of three hidden layers with 30 neurons each and was trained for 5000 epochs using the L-BFGS optimizer. The reported results showed that the PINN generalized better than an ANN when the excitation voltage of the test data differed from that of the training data. For the three-hidden-layer, 30-neuron configuration with a physics-loss weight of one, the reported relative errors were approximately 0.11, 0.17 and 0.39 for 1.1 UN, UN and 0.9 UN, respectively, whereas the corresponding ANN errors were 0.17, 0.34 and 0.76. However, the authors also emphasized that the prediction error remains influenced by the physical parameters Kσ and Ks, and that the method is particularly suitable when mechanical stress is kept constant while the excitation voltage varies only slightly. Furthermore, the target of the model is magnetostriction rather than the direct reconstruction of complete BH hysteresis loops, and the experimental study considers grain-oriented steel under tensile loading rather than non-oriented steel subjected to both tensile and compressive stresses and multiple material orientations.

A complementary recent direction concerns the use of deep learning to accelerate JA parameter identification. She et al. [10] proposed a physics-guided Kolmogorov–Arnold Network (KAN-EfficientNet) architecture for fast inversion of JA parameters. Physical constraints from the inverse JA model were embedded in the learning framework, while a KANLinear layer was used to improve representation and reduce model complexity. The method was evaluated using numerical/simulation data together with experimental verification and achieved parameter-estimation errors below 2.41% with a reported calculation time of approximately 0.3 s. This study demonstrates that physics-guided learning can substantially improve the speed and robustness of JA parameter inversion compared with conventional iterative optimization. However, its objective is parameter estimation rather than direct forward prediction of stress- and frequency-dependent hysteresis. It therefore does not address the use of a JA-Sablik magneto-mechanical baseline followed by deep residual correction across multiple sample orientations and mechanical loading conditions.

Taken together, these studies show a clear progression from physically interpretable hysteresis models toward hybrid and physics-guided learning strategies. Table 1 summarizes the main contribution, advantage and remaining limitation of the four recent approaches that are most closely related to the present work. The comparison indicates that dynamic hybridization [7], explainable JA-based neural modelling [8], physics-informed magnetostriction prediction [9] and physics-guided JA parameter inversion [10] each address an important part of the overall problem. However, none of these studies simultaneously combines a stress-aware JA-Sablik physical baseline, multi-condition non-oriented electrical-steel measurements, frequency and orientation information, and deep sequential residual learning for complete BH loop correction. This unresolved combination defines the methodological space addressed in the present study.

### 1.2. Research Gaps

The analysis above and Table 1 show that the recent literature has made substantial progress in physical hysteresis modelling, explainable neural modelling, physics-informed learning and parameter identification. At the same time, the contributions remain largely complementary rather than fully integrated. The following research gaps are therefore identified for magneto-mechanical hysteresis prediction in non-oriented electrical steel.

1. First, interpretability and predictive flexibility are still difficult to achieve simultaneously. Pure neural hysteresis models can learn complex nonlinear and rate-dependent mappings, but the learned parameters generally have no direct magnetic meaning and their reliability depends strongly on the coverage of the training data. Ni et al. [8] reduced this limitation by embedding the JA structure in an explainable JANN-NARX architecture, while Hong et al. [9] imposed physical information through a PINN residual formulation. Nevertheless, neither approach addresses complete stress-dependent BH hysteresis of non-oriented electrical steel over multiple orientations and frequencies.
2. Second, physically based JA and JA-Sablik models preserve meaningful magnetic and magneto-mechanical parameters, but their accuracy depends on parameter identification and on how completely the analytical equations represent the relevant mechanisms. Dynamic excitation, anisotropy, mechanical stress and magnetostriction can interact, and a single parameter set may not reproduce every measured loop with the same accuracy. The recent work of She et al. [10] demonstrates that physics-guided deep learning can greatly accelerate JA parameter inversion, but parameter inversion alone does not remove structural modelling error in the forward hysteresis model.
3. Third, existing hybrid approaches generally solve only part of the combined problem. Li et al. [7] successfully couple JA and Preisach models for dynamic hysteresis and core-loss prediction, but do not consider external mechanical stress. Hong et al. [9] incorporate stress in a physics-informed magnetostriction model, but the target variable is magnetostriction rather than the complete BH trajectory. Ni et al. [8] address explainable rate-dependent hysteresis, but for piezoelectric systems. Consequently, the simultaneous integration of a Sablik stress-aware physical model with a sequential residual learner for full magnetic hysteresis remains insufficiently investigated.
4. Finally, validation across the combined experimental factors relevant to electrical steel applications remains limited. The present dataset contains non-oriented electrical steel sheets cut along RD, DD and TD directions and measured under tensile, compressive and zero stress conditions at different excitation frequencies and different peak field. A framework is therefore needed that can exploit these factors explicitly while preserving physical structure and learning only the discrepancy that remains between the analytical JA-Sablik prediction and the measured B-H loop.

These gaps motivate a hybrid strategy in which the JA-Sablik formulation remains the primary physical representation, while deep learning is restricted to residual correction. Such a formulation avoids discarding known magneto-mechanical physics, yet provides sufficient flexibility to capture nonlinear effects that remain unresolved by the analytical model under combined stress, frequency and sample-orientation variations.

### 1.3. Contributions and Paper Organization

Motivated by the research gaps identified above, this paper develops a hybrid Jiles-Atherton-Sablik and CNN-BiLSTM framework for magneto-mechanical hysteresis prediction in non-oriented electrical steel. The proposed architecture differs from the reviewed approaches by combining a stress-aware physical baseline with residual sequential learning and by validating the complete workflow across experimentally measured conditions covering different orientations, applied stress levels, excitation frequencies, and magnetic excitation amplitudes. The main contributions are as follows:

1. A hybrid JA-Sablik based Convolutional Neural Network and Bidirectional Long-Short Term Memory (CNN-BiLSTM) architecture is developed in which the analytical JA-Sablik model generates the baseline B-H response and the neural network learns only the residual error between the physical prediction and the experiment. This division preserves the physical role of the hysteresis model while assigning the data-driven component a clearly defined correction task.
2. The four JA parameters are calibrated through optimization while Sablik-related quantities such as saturation magnetization, magnetostriction and characteristic stress are retained as material-based physical parameters. This reduces the risk of allowing the data-driven stage to absorb parameters that should remain physically interpretable.
3. The framework is validated using non-oriented electrical-steel measurements that include RD, DD and TD sample orientations, tensile/compressive/zero-stress states and multiple excitation frequencies. This multi-condition validation directly targets the coupled operating factors that are only partially addressed in the reviewed studies.
4. A convolutional-recurrent residual learner is employed so that local waveform features are extracted by the CNN layers while the BiLSTM layers capture sequential hysteresis memory. The resulting hybrid model is therefore designed to correct unmodelled nonlinear, dynamic and magneto-mechanical effects without replacing the JA-Sablik physical core.

The remainder of this paper is organized as follows. Section 2 presents the experimental materials, measurement procedure and dataset structure. Section 3 introduces the mathematical formulation of the JA-Sablik model, the parameter-calibration procedure and the CNN-BiLSTM residual-learning architecture. Section 4 presents and discusses the experimental and modelling results, including the comparison between the physics-only and hybrid predictions. Finally, Section 5 summarizes the main comparisons and limitations of the study while Section 6 devoted for the conclusions and outlines directions for future work.

## 2. Materials

This section describes the experimental setup, sample characteristics, measurement procedures, and dataset structure used in this work. The data processing methodology, dataset organization, representative experimental results, and measurement limitations are presented in the following sections.

### 2.1. Experimental Setup and Samples

The experimental database used in this work is obtained from [14,15] and consists of non-oriented Fe–3%Si electrical steel sheets measured under magneto-mechanical loading. In their work [14,15], three samples were evaluated, cut at orientations of 0° (RD: rolling direction), 45° (DD: diagonal direction), and 90° (TD: transverse direction) relative to the rolling direction to investigate the anisotropic magnetic behavior of the material. The sample dimensions, summarized in Table 2, were obtained from an average of at least three measurement sets to minimize dimensional uncertainties. Each sample has a cross-sectional area of approximately 5 × 10^−5^ m^2^, with variations primarily due to differences in thickness and width resulting from the cutting process. The density values range from 7845 to 8135 kg/m^3^, which are consistent with typical values for iron-silicon electrical steel alloys.

**Figure 1:**
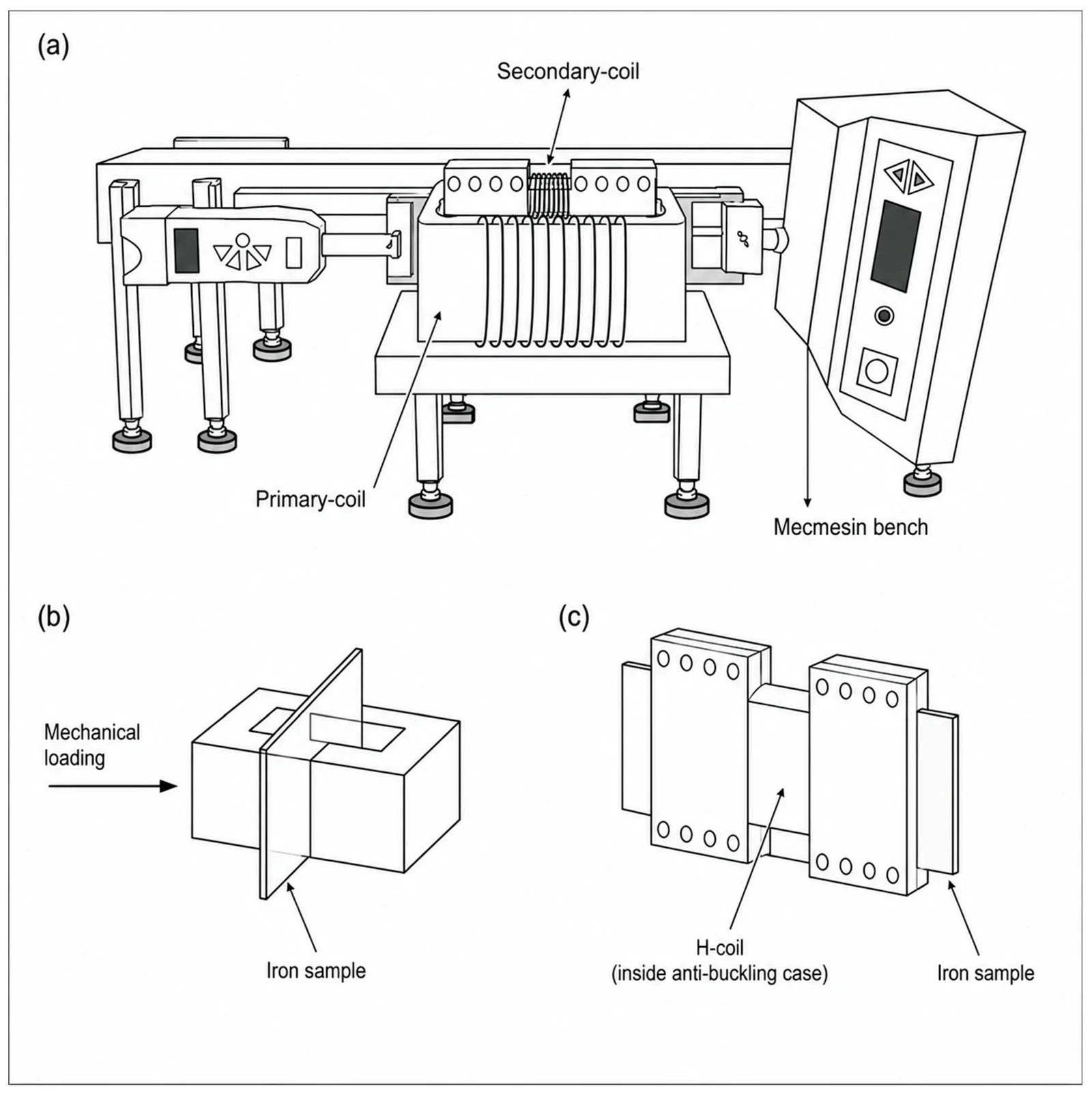
Schematic representation of the modified SST experimental setup and sample configurations: (**a**) modified SST bench; (**b**) mechanical loading configuration of the iron sample; (**c**) anti-buckling case with integrated H-coil measurement system. The schematic is adapted from [14,15].

As presented in Figure 1, the experimental bench combines a modified Single Sheet Tester (SST), a Mecmesin^®^ (Mecmesin, Slinfold, UK) universal testing machine for mechanical loading, and H-coil sensors for direct magnetic field measurement. The system is capable of simultaneously applying uniaxial mechanical stress and controlled magnetic flux to the samples. Mechanical loads include tensile and compressive stresses ranging from 5 to 20 MPa, applied in 5 MPa increments. Magnetic field measurement is performed using three H-coil sensors with cross-sectional areas on the order of 5 × 10^−5^ m^2^, yielding induced voltages from 0.7 µV to 20 µV RMS at 1 Hz, amplified with a gain factor of 1035. Data acquisition is conducted using a National Instruments^®^ NI-6212 (National Instruments Corporation, Austin, TX, USA) board controlled via a LabVIEW virtual instrument (National Instruments Corporation, Austin, TX, USA).

The experimental procedure consists of two phases, starting with the mechanical stress configuration, followed by the magnetic excitation. Uniaxial tensile and compressive stresses of 5, 10, 15, and 20 MPa, as well as a no-stress condition (0 MPa), are applied. The applied force is regulated by adjusting the displacement of the Mecmesin^®^ universal testing machine. An anti-buckling fixture is incorporated to prevent bending of the steel sheets under compressive stress. Signal waveforms are monitored using an oscilloscope to ensure waveform fidelity throughout the test. For magnetic excitation, sinusoidal induction waveforms are imposed at 1 Hz, 50 Hz, and 100 Hz, with peak induction levels ranging from 0.05 T to 1.5 T in 0.05 T steps. The samples are demagnetized before each set of experiments to ensure reproducible initial conditions.

### 2.2. Data Processing

Signal acquisition includes the excitation voltage, excitation current (using a 100 mV/A probe), secondary winding voltage, and the three amplified H-coil sensor voltages. Data collection and initial waveform inspection are managed using a LabVIEW Virtual Instrument. Magnetic induction *B*(*t*) is calculated by integrating the secondary winding voltage *v*_s_ as shown in Equation (1), where *N*_s_ is the number of turns (375 turns) and *S* is the cross-sectional area of the sample [16,17] and then we use the absolute value of B (i.e., |*B*|).

(1) $$B(t)=\int_{0}^{t}[-1/(N_{s}S)]v_{s}(t^{\prime }){\mathrm{dt}}^{\prime }$$

Magnetic field strength *H*(*t*) is determined by integrating the voltages *v_H_* from the three H-coil sensors at known distances, as shown in Equation (2), where *N_H_* (2000 turns) and *S_H_* (≈3.2 × 10^−5^ mm^2^) are the number of turns and cross-sectional area of each H-coil winding, and *μ*_0_ is the magnetic permeability of free space (4π × 10^−7^ H/m) [16,17].

(2) $$H_{\mathrm{coil}}(t)=1/(N_{H}S_{H}\mu_{0})\int_{0}^{t}v_{H}(t^{\prime }){\mathrm{dt}}^{\prime }$$

The magnetic field at the surface of the sample, denoted as *H_extrap_* in the dataset, is determined using an extrapolation procedure applied to measurements from the three H-coil sensors, based on the principle of tangential field continuity across media with differing magnetic permeabilities. The magnetic energy loss per unit volume (J/m^3^) is estimated from the area of the BH loop, numerically calculated using both ∫H dB and ∫*B dH* approaches. Integrations are performed using the trapezoidal rule across discrete data points, and the results are averaged to minimize numerical discrepancies. The energy loss (J/kg) is calculated by dividing the volumetric energy loss by the sample density.

### 2.3. Dataset Structure

The dataset is organized in a hierarchical directory structure designed to facilitate efficient data retrieval by orientation, frequency, and stress. The main directory contains three subfolders corresponding to sample orientation RD, DD, and TD. Within each, data is further organized by test frequency (1 Hz, 50 Hz, and 100 Hz). Each frequency folder contains two types of data: BH loops (instantaneous magnetic induction and field strength) and loss curves (magnetic loss values in J/kg). The BH loop folders are subdivided into 9 subfolders representing specific mechanical stress levels and types, indicated by prefixes “T” (tensile) and “C” (compressive) followed by the stress value in MPa. Each BH loop file is a CSV with 10,000 time-series data points per full cycle, where the first column is the magnetic field strength *H_extrap_*(*t*) and the second column is the magnetic induction *B*(*t*). File names are standardized to include the peak induction, frequency, and variables. Similarly, loss curve folders contain nine CSV files, each reporting magnetic losses (J/kg) as a function of peak induction.

### 2.4. Representative Experimental Data

**Figure 2:**
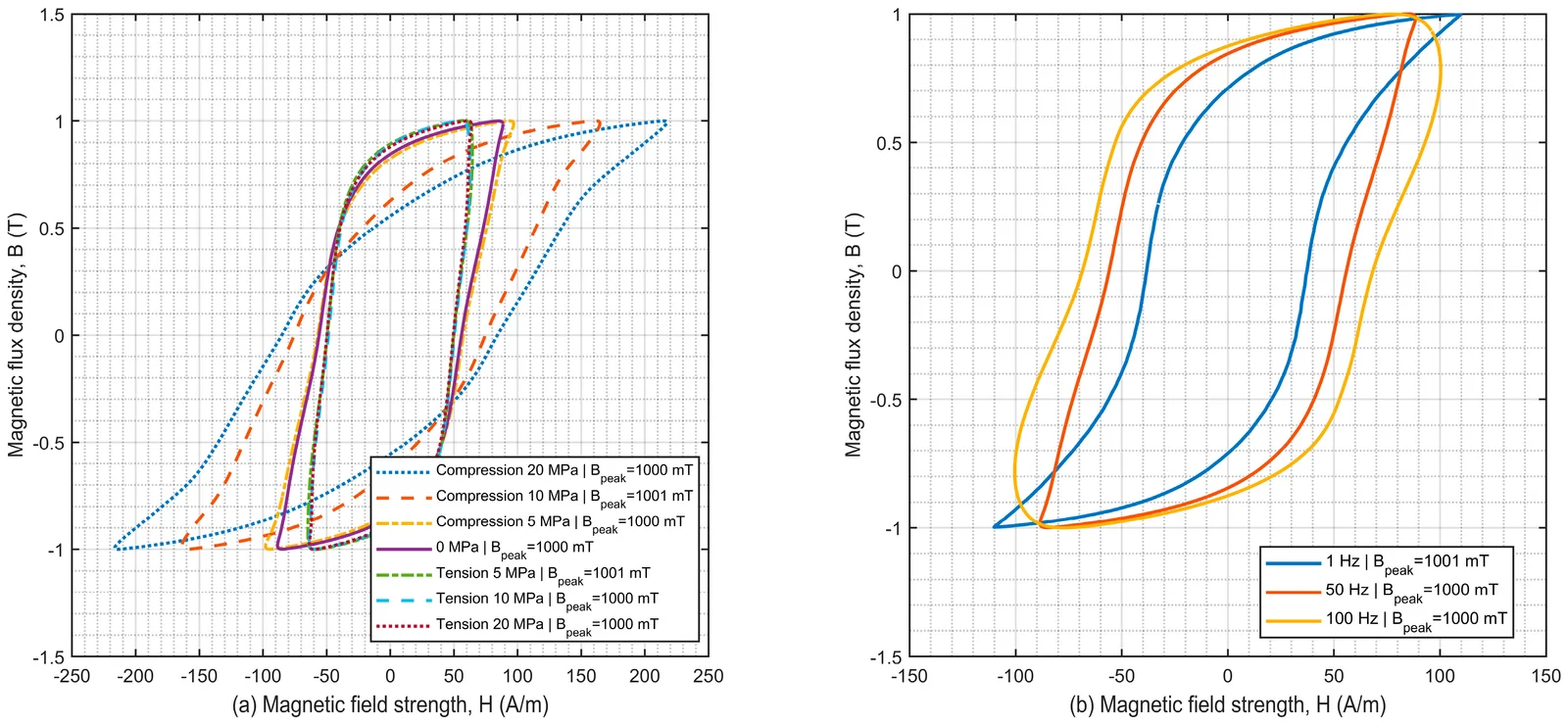
Representative experimental B-H hysteresis loops of TD samples: (**a**) Effect of mechanical stress at 50 Hz; (**b**) Effect of excitation frequency at 0 MPa.

**Figure 3:**
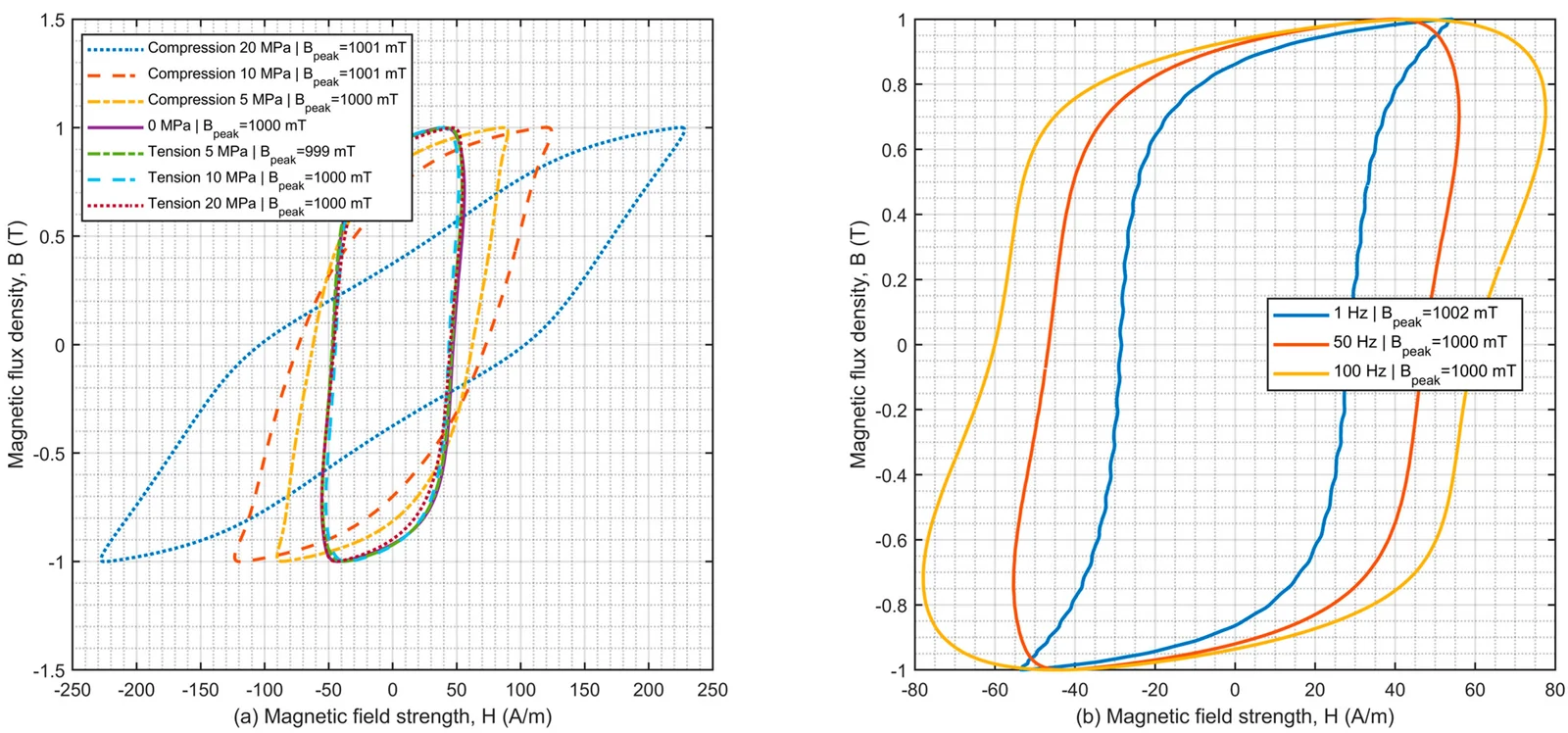
Representative experimental BH hysteresis loops of RD samples: (**a**) Effect of mechanical stress at 50 Hz; (**b**) Effect of excitation frequency at 0 MPa.

**Figure 4:**
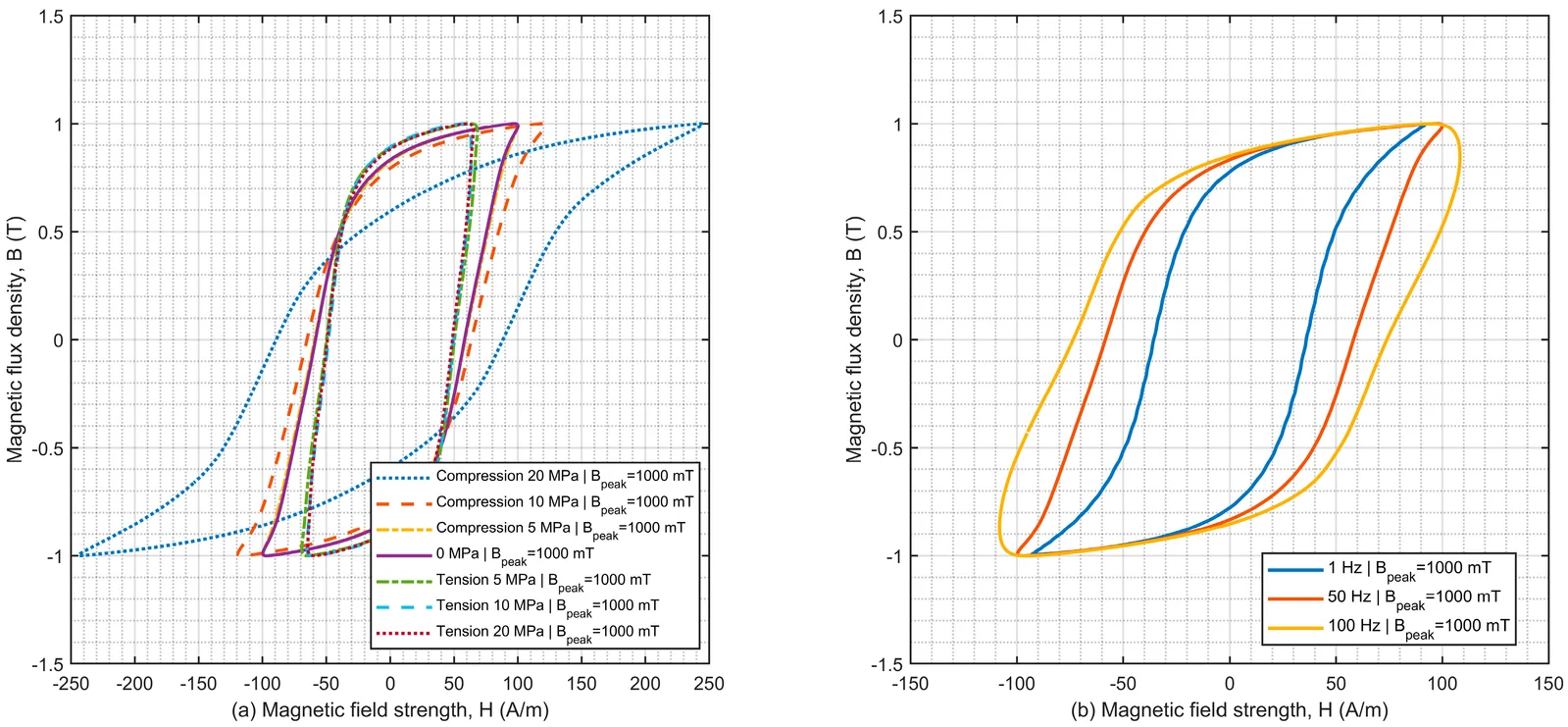
Representative experimental B-H hysteresis loops of DD samples: (**a**) Effect of mechanical stress at 50 Hz; (**b**) Effect of excitation frequency at 0 MPa.

**Figure 5:**
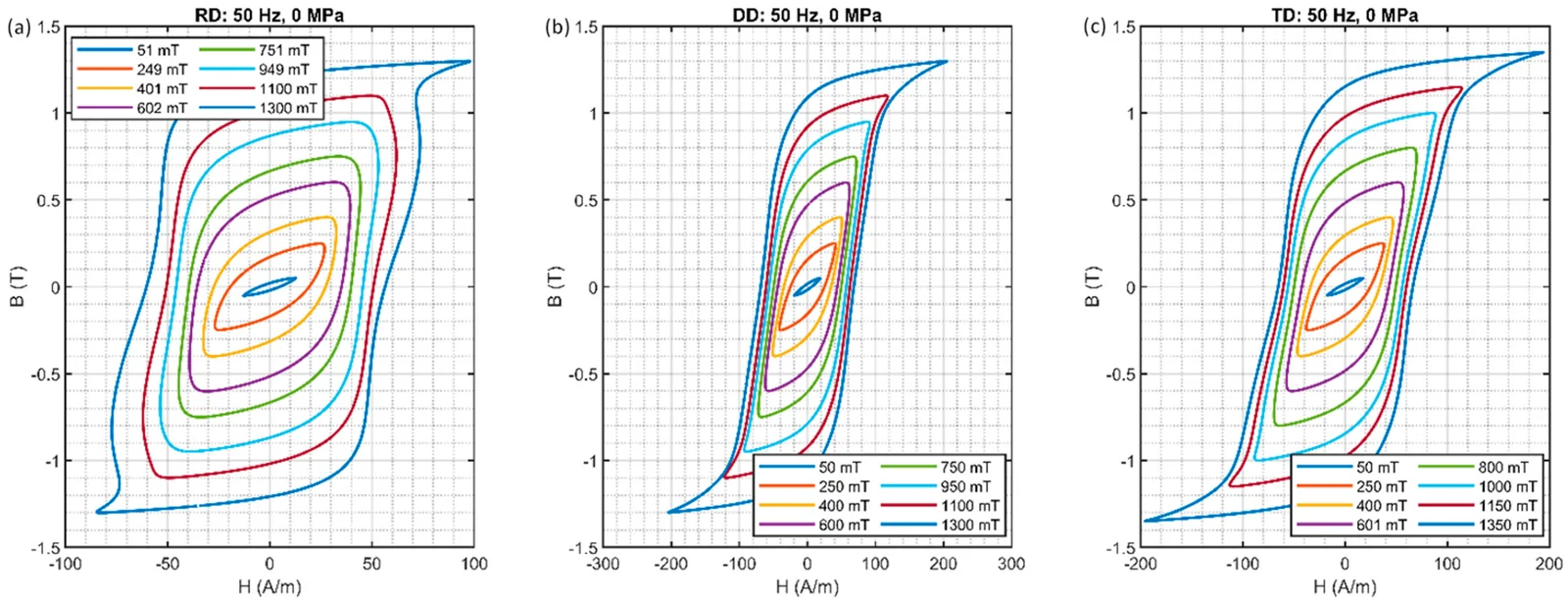
Experimental hysteresis loops at multiple peak induction levels: (**a**) RD, 50 Hz, 0 MPa; (**b**) DD, 50 Hz, 0 MPa; (**c**) TD, 50 Hz, 0 MPa.

**Figure 6:**
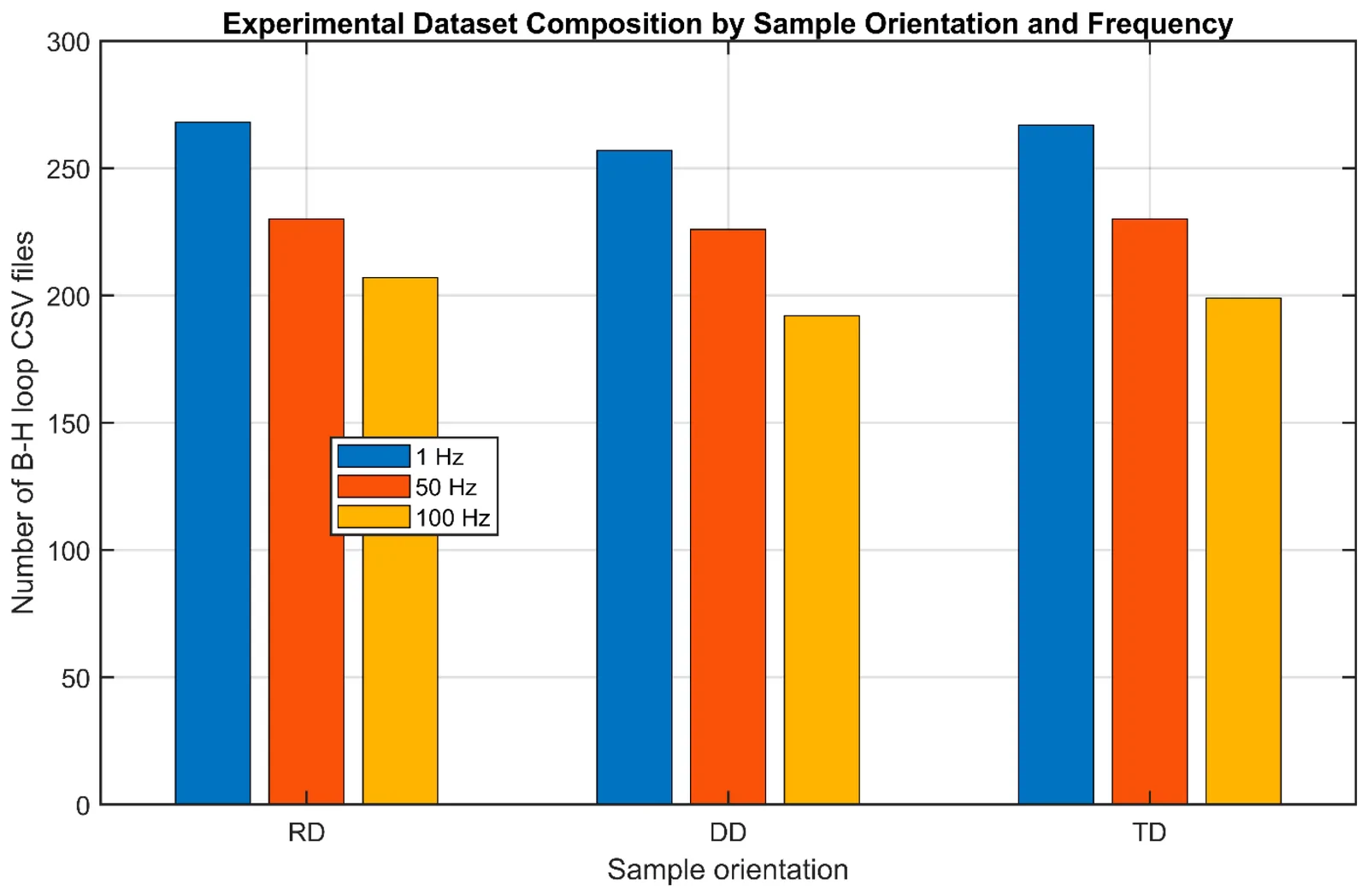
Experimental dataset composition by sample orientation and frequency.

The complete dataset contains approximately 83 MB of experimental measurements. Figure 2, Figure 3, Figure 4, Figure 5 and Figure 6 provide representative B–H loops for the three sample orientations and highlight how the magnetic response changes with mechanical stress, excitation frequency, and peak induction.

Figure 2 presents representative hysteresis loops for the TD sample. In Figure 2a, recorded at 50 Hz, the effect of mechanical stress is clearly visible. Compressive loading from −20 MPa to −5 MPa generally produces wider loops and lower permeability, whereas tensile loading from +5 MPa to +20 MPa tends to narrow the loops and increase permeability. This behavior is consistent with the inverse magnetostrictive, or Villari, effect. Figure 2b illustrates the influence of excitation frequency at zero applied stress. As the frequency increases from 1 Hz to 100 Hz, the hysteresis loop becomes broader and its enclosed area increases, reflecting the growing contribution of dynamic losses. Despite this increase in loop area, the overall shape of the hysteresis response remains broadly similar. These observations confirm that the magnetic behavior of the TD sample is strongly influenced by both mechanical loading and excitation frequency.

Figure 3 shows representative BH hysteresis loops for the RD sample and highlights the influence of both mechanical stress and excitation frequency. In Figure 3a, measured at 50 Hz, compressive stress leads to broader hysteresis loops and a reduction in permeability, whereas tensile stress produces narrower loops and higher permeability. Compared with the other sample orientations, the RD example shows the highest permeability, which can be attributed to the more favorable alignment of the magnetic easy axes with the rolling direction. Figure 3b illustrates the effect of frequency at zero applied stress. As the excitation frequency increases from 1 Hz to 100 Hz, the loop area becomes larger, indicating an increase in dynamic magnetic losses. The curves are shown at approximately the same peak induction level, around 1000 mT, allowing a direct comparison between operating conditions. Overall, the results show that the magnetic response of the RD sample is strongly affected by both mechanical loading and excitation frequency.

Figure 4 presents representative BH hysteresis loops for the DD sample and illustrates how its magnetic response changes with mechanical stress and excitation frequency. In Figure 4a, measured at 50 Hz, compressive stress produces broader hysteresis loops and lower permeability, whereas tensile stress leads to narrower loops and higher permeability, in agreement with the Villari effect [18,19]. The DD sample shows an intermediate magnetic response compared with the RD and TD orientations. Figure 4b highlights the influence of frequency under zero applied stress. As the excitation frequency increases from 1 Hz to 100 Hz, the hysteresis loop area becomes larger, reflecting the greater contribution of dynamic losses. The curves are compared at similar peak induction levels of approximately 1000 mT. Overall, these results show that the magnetic behavior of the DD sample is noticeably influenced by both mechanical loading and excitation frequency.

Figure 5 illustrates the evolution of the experimental BH hysteresis loops as the peak induction increases for the three sample orientations under zero applied stress at 50 Hz. Figure 5a–c correspond to the RD, DD, and TD samples, respectively. The nested loops cover peak induction levels from 0.05 T to 1.5 T, with increments of 0.05 T, providing a detailed view of the magnetization process from low excitation to near-saturation conditions.

As the induction level increases, the loops progressively expand and become more sheared, reflecting the nonlinear approach to magnetic saturation. Clear differences can also be observed between the three orientations. The RD sample shows the highest permeability and generally narrower loops, whereas the TD sample exhibits lower permeability and wider loops. The DD sample presents an intermediate response, consistent with its 45° orientation relative to the rolling direction. These differences highlight the magnetic anisotropy of the material and show how the hysteresis response evolves over a broad range of excitation levels.

Figure 6 provides an overview of the experimental dataset by showing the number of available BH loop files for the RD, DD, and TD samples at 1, 50, and 100 Hz. The measurements are distributed fairly evenly across the different orientations and excitation frequencies, which indicates that the main operating conditions are well represented. The RD sample contains slightly more measurements in some cases, particularly at 1 Hz, whereas the DD and TD samples show similar distributions across the investigated frequencies. This balanced coverage is suitable for training and evaluating the proposed hybrid model under different material orientations and excitation conditions.

The ability to achieve higher peak magnetic induction levels was constrained by the limitations of the experimental setup, particularly under compressive mechanical stress conditions where increased magnetic losses and power demand restricted the applied excitation. Table 3 presents the maximum peak induction values achieved for each sample under different frequency and mechanical stress conditions. Under compressive stress, the maximum achievable induction decreased due to increased losses, especially at higher frequencies (100 Hz) where the induction limit dropped to as low as 0.9 T for some conditions.

The dataset provides comprehensive coverage of the experimental parameter space and is suitable for developing and validating models of magneto-mechanical coupling. The hierarchical structure enables efficient data retrieval and supports research on stress-dependent magnetic behaviour in electrical steels.

## 3. Methods

**Figure 7:**
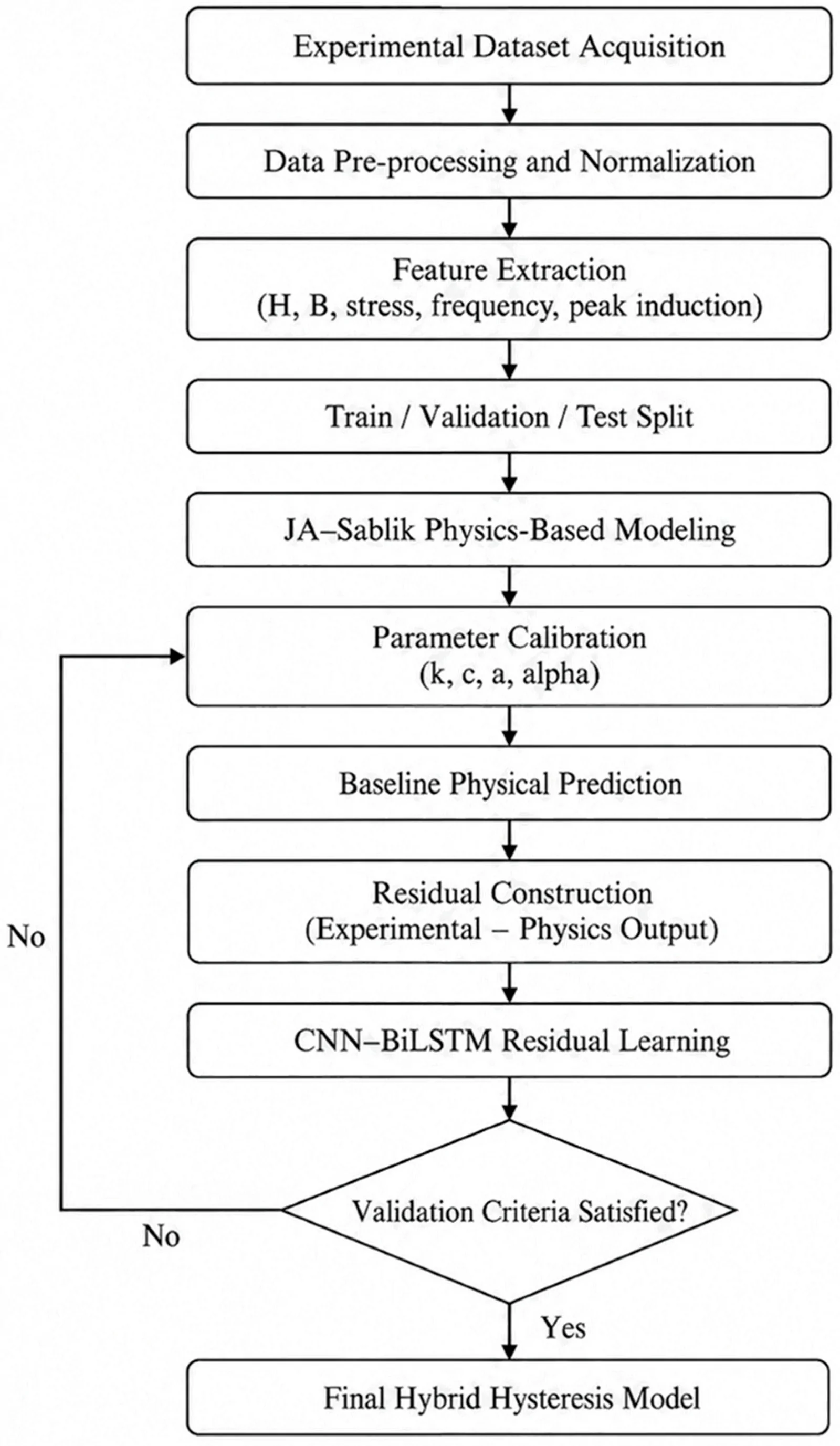
Schematic of the hybrid modelling framework combining Jiles–Atherton–Sablik physics with CNN-BiLSTM residual correction.

This section describes the hybrid modelling framework combining a physics-based Jiles–Atherton–Sablik model with a deep residual correction network. The overall methodology follows a cascaded architecture where the physical model first generates a baseline prediction of the BH loop, and the neural network subsequently learns the residual error between the experimental measurements and the physics-based prediction. A schematic overview of the proposed approach is presented in Figure 7. The experimental inputs (H, frequency, stress, orientation) feed the physical model, producing a baseline JA prediction. The residual network learns the error Δ*B* between the experimental measurement and the physical prediction. The hybrid output combines both contributions.

### 3.1. Jiles–Atherton Hysteresis Model

The Jiles–Atherton (JA) formulation represents the total magnetization as the sum of reversible and irreversible contributions [20,21,22]. The notation and equations for the JA and Sablik models follow [23,24]. The total magnetization and reversible component are written as

*M* = *M_rev_* + *M_irr_*. (3)

*M_rev_* = *c*(*M_an_* − *M_irr_*). (4)

The irreversible component and the anhysteretic magnetization follow the reference formulation

(5) $$M_{\mathrm{irr}}=M_{\mathrm{an}}-k\delta \frac{dM_{\mathrm{irr}}}{dH_{e}}.$$

(6) $$M_{\mathrm{an}}=M_{s}\left[\coth\left(\frac{H_{e}}{a}\right)-\frac{a}{H_{e}}\right].$$

At fixed temperature, the rate of change of magnetization depends on both the differential magnetic susceptibility and the magnetization change induced by stress. Accordingly,

(7) $$\frac{\mathrm{dM}}{\mathrm{dt}}=\frac{\mathrm{dM}}{\mathrm{dH}}\frac{\mathrm{dH}}{\mathrm{dt}}+\frac{\mathrm{dM}}{\mathrm{d\sigma}}\frac{\mathrm{d\sigma}}{\mathrm{dt}}.$$

The stress derivative is expressed as

(8) $$\frac{\mathrm{dM}}{\mathrm{d\sigma}}=\frac{\sigma }{\mathrm{Y\xi}}\left(M_{\mathrm{an}}-M\right)+c\frac{dM_{\mathrm{an}}}{\mathrm{d\sigma}}.$$

Here, *M_s_* is the saturation magnetization, *k* is the average density of pinning sites, *c* is the reversibility coefficient, *a* is the effective-field scaling parameter, *Y* is Young’s modulus, and *ξ* is the coefficient governing conversion of elastic-energy variation into irreversible magnetization change. The directional parameter *δ* distinguishes increasing and decreasing field branches.

### 3.2. Sablik Magneto-Mechanical Coupling

The Sablik extension introduces the influence of mechanical stress through the stress effective field and the stress-demagnetization contribution [20,21,22]. The effective field is written as

*H_e_* = *H_a_* − *H_d_* + *αM* − *D_σ_M* + *H_σ_*. (9)

The stress effective field is proportional to the magnetization derivative of the bulk magnetostriction:

(10) $$H_{\sigma }=\frac{3\sigma }{2\mu_{0}}\frac{\mathrm{d\lambda}}{\mathrm{dM}}.$$

Differentiation of the effective-field expression leads to the stress-dependent interaction coefficient

(11) $$\alpha_{e}\left(\sigma \right)=\alpha -D_{\sigma }+\frac{3\sigma }{2\mu_{0}}\frac{d^{2}\lambda }{dM_{a}^{2}}.$$

The stress-demagnetization term is

(12) $$D_{\sigma }=\frac{3\lambda_{s}}{\mu_{0}M_{s}^{2}}\sigma =D_{s}\sigma .$$

To represent the magnetostrictive response, the Sablik relation adopted by the reference model is

(13) $$\lambda =-\frac{b\left(1+\nu \right)}{Y}\left\{{\left[1+\frac{9Y}{2b^{2}{\left(1+\nu \right)}^{2}}\phi_{\mathrm{mag}}\left(M_{s}\right)\right]}^{1/2}-{\left[1+\frac{9Y}{2b^{2}{\left(1+\nu \right)}^{2}}\left(\phi_{\mathrm{mag}}\left(M_{s}\right)-\phi_{\mathrm{mag}}\left(M\right)\right)\right]}^{1/2}\right\}.$$

The magnetic-energy terms and the domain-coupling constant are defined by

(14) $$\phi_{\mathrm{mag}}=U_{\mathrm{mag}}+\phi_{\mathrm{hys}}\;U_{\mathrm{mag}}=\frac{1}{2}\hat{\alpha }\mu_{0}M^{2}\;\phi_{\mathrm{hys}}=\frac{1}{2}\alpha^{\prime \prime }\mu_{0}{\left(M_{a}-M\right)}^{2}-\alpha^{\prime }\mu_{0}\left(M_{a}-M\right)H\;\hat{\alpha }=\frac{21}{4}\frac{Y}{\mu_{0}}{\left(\frac{\lambda_{s}}{M_{s}}\right)}^{2}.$$

For the isotropic approximation, the elastic quantities are related through

(15) $$\nu =\frac{c_{12}}{c_{11}+c_{12}}.$$

*Y* = *c*_11_ − 2*c*_12_*ν*. (16)

$$c_{44}=\frac{c_{11}-c_{12}}{2}$$

(17) $$b=\left(\frac{2}{5}\lambda_{100}+\frac{3}{5}\lambda_{111}\right)c_{44}.$$

The material constants associated with the theoretical Sablik magnetostriction formulation for NO Fe-Si 3% are summarized below. The hysteretic terms $\alpha^{\prime }$ and $\alpha^{\prime \prime }$ control the cycling behaviour of the magnetostriction loop.
**Parameter****Value***c*_11_202 GPa*c*_12_122 GPa*λ*_100_23 × 10^−6^*λ*_111_−4.5 × 10^−6^$\alpha^{\prime }$7.5 × 10^6^$\alpha^{\prime \prime }$3.5 × 10^5^

### 3.3. Dynamic Loss Model

To extend the magnetization model to frequency-dependent operation, the eddy-current contribution is incorporated through two coefficients representing anomalous/excess and classical eddy-current effects [20,21,22]. The coefficients are:

(18) $$K_{1}={\left(\frac{Gd_{1}wH_{0}\mu_{0}}{\rho }\right)}^{1/2},\;\;K_{2}=\frac{\mu_{0}^{2}d_{1}^{2}}{2\rho \beta }.$$

Here, *d*_1_ is the sheet thickness, *ρ* is the electrical resistivity, *β* is the geometry factor, *G* is a dimensionless constant, *w* is the sheet width, and *H*_0_ is the fluctuating internal field experienced by the domain walls. Incorporating the stress-dependent interaction coefficient into the differential-susceptibility equation gives

(19) $$\left[k\delta -\alpha_{e}\left(\sigma \right)\left(M_{\mathrm{an}}-M+k\delta c\frac{dM_{\mathrm{an}}}{dH_{e}}\right)\right]\frac{\mathrm{dM}}{\mathrm{dH}}-\left(M_{\mathrm{an}}-M+k\delta c\frac{dM_{\mathrm{an}}}{dH_{e}}\right)\;+K_{2}\frac{\mathrm{dH}}{\mathrm{dt}}{\left(\frac{\mathrm{dM}}{\mathrm{dH}}\right)}^{2}+K_{1}{\left(\frac{\mathrm{dH}}{\mathrm{dt}}\right)}^{1/2}{\left(\frac{\mathrm{dM}}{\mathrm{dH}}\right)}^{3/2}=0.$$

Equation (19) therefore represents the stress- and frequency-dependent hysteresis law *M*(*H*,*σ*,*f*). In the numerical workflow, pure JA, JA-Sablik, and dynamic JA-Sablik stages are evaluated separately for diagnostic comparison, while the selected physics baseline is subsequently supplied to the residual-learning stage.

### 3.4. Parameter Optimization

The four JA parameters *k*, *c*, *a*, and *α* are calibrated using Bayesian optimization [25]. Consistent with the MATLAB (V.23.2.0.2515942 (R2023b) Update 7) implementation, calibration is performed on representative low-frequency, near-zero-stress loops, while stress coupling and dynamic losses are disabled during this first stage. The optimization vector is

**θ** = [*k*, *c*, *a*, *α*]*^T^*. (20)

Rather than minimizing only a pointwise error, the implemented calibration objective combines the normalized trajectory error with penalties on important loop characteristics:

(21) $$J\left(\theta \right)=\mathrm{NRMSE}+0.15\;e_{H_{c}}+0.15\;e_{B_{r}}+0.10\;e_{A}.$$

The normalized trajectory error is

(22) $$\mathrm{NRMSE}=\frac{\mathrm{RMSE}}{\max\left(\max\left|B_{\exp}\right|,10^{-6}\right)}.$$

The characteristic-loop errors are

(23a) $$e_{H_{c}}=\frac{\left|H_{c,\mathrm{sim}}-H_{c,\exp}\right|}{\max\left(\left|H_{c,\exp}\right|,1\right)}.$$

(23b) $$e_{B_{r}}=\frac{\left|B_{r,\mathrm{sim}}-B_{r,\exp}\right|}{\max\left(\left|B_{r,\exp}\right|,0.01\right)}.$$

(23c) $$e_{A}=\frac{\left|A_{\mathrm{sim}}-A_{\exp}\right|}{\max\left(\left|A_{\exp}\right|,10^{-6}\right)}.$$

The optimized parameter vector is therefore

θ^*^ = arg min_**θ**_ *J*(**θ**). (24)

The search intervals implemented in the code are *k* ∈ [10, 150] A/m, *c* ∈ [0.01, 0.95], *a* ∈ [50, 200] A/m, and *α* ∈ [10^−4^, 5 × 10^−3^]. The saturation magnetization and saturation magnetostriction are treated as fixed physical properties, whereas the four JA parameters are calibrated.

### 3.5. Residual Correction Network

The deep residual correction is implemented using a Convolutional Recurrent Residual Network (CRRN) [25,26,27,28,29]. The target learned by the network is the difference between the experimental induction and the selected physics baseline:

*ΔB*(*t*) = *B_exp_*(*t*) − *B_base_*(*t*). (25)

**Figure 8:**
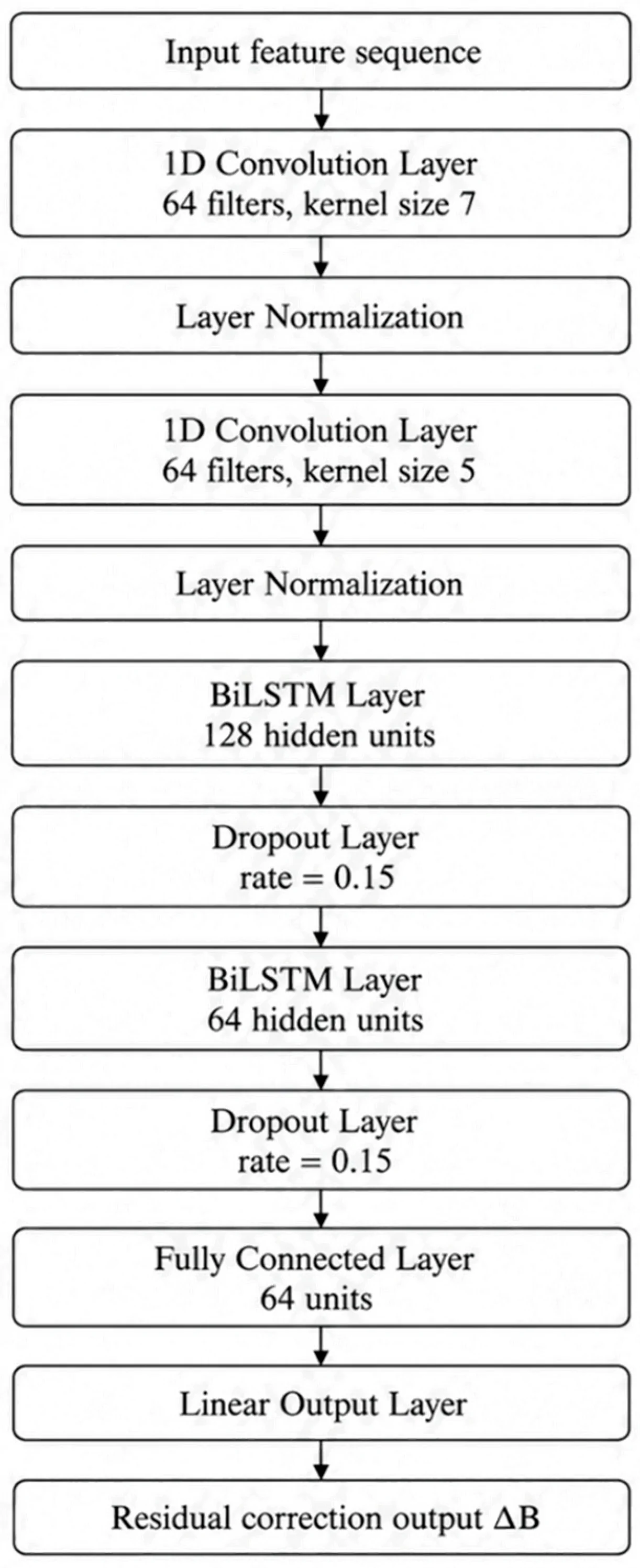
Architecture of the Convolutional Recurrent Residual Network (CRRN).

As shown in Figure 8, the CRRN begins with two one-dimensional convolutional layers, each containing 64 filters with kernel sizes of 7 and 5. Layer normalization is applied after each convolutional layer. The extracted local features are then processed by two BiLSTM layers containing 128 and 64 hidden units, respectively. Dropout with a rate of 0.15 is applied to improve generalization, followed by a fully connected layer with 64 units and a linear output layer that predicts the residual correction.

The input sequence implemented in MATLAB contains nine features:

(26) $$x\left(t\right)={\left[\tilde{H}\left(t\right),\;{\tilde{B}}_{\mathrm{base}}\left(t\right),\;\tilde{\dot{H}}\left(t\right),\;d\left(t\right),\;\tilde{\log_{10}f},\;\tilde{\sigma },\;{\tilde{B}}_{\mathrm{peak}},\;\sin\left(2\pi \varphi \right),\;\cos\left(2\pi \varphi \right)\right]}^{T}.$$

Here, the tilde denotes standardization using statistics fitted only on the training set, *d*(*t*) represents the direction of field variation, and *φ* is the normalized cycle phase.

(27) $$L=\frac{1}{N}\sum_{i=1}^{N}{\left({\tilde{\Delta B}}_{i}-{\hat{\tilde{\Delta B}}}_{i}\right)}^{2}.$$

Training uses the Adam optimizer with an initial learning rate of 1 × 10^−3^ and early stopping based on the validation loss. After reversing the residual normalization, the final hybrid prediction is

(28) $$B_{\mathrm{hybrid}}\left(t\right)=B_{\mathrm{base}}\left(t\right)+\hat{\Delta B}\left(t\right).$$

Before the final test metrics are computed, the implementation applies a smooth endpoint correction to the predicted cycle so that the first and last induction values coincide, preventing an artificial opening of the reconstructed hysteresis loop. This preserves the physics-based baseline while correcting the residual trajectory learned by the network. The complete methodology is summarized in the pseudocode provided in Algorithm 1.
**Algorithm 1.** Hybrid JA-Sablik + CRRN modelling workflow.
**Input:** Experimental loop set $D={\left\{H_{i}\left(t\right),B_{i}^{\exp\left(t\right)},f_{i},\sigma_{i},p_{i},s_{i}\right\}}_{i=1}^{N_{L}}$; fixed material parameters *μ*_0_, *M_s_*, *λ_s_*, *σ*_0_; initial JA parameters; physics and CRRN configuration. **Output:** Optimized JA parameters $\theta_{\mathrm{JA}}^{*};$ trained residual network $N_{\psi^{*}}$; hybrid predictions $B_{i}^{\mathrm{hyb}}\left(t\right)$; unseen-test MAE, RMSE, and *R*^2^.1**Initialize** the fixed material constants and numerical settings; set the common sequence length *L*.2Scan the experimental database, load the eligible *B*–*H* loops, and reject incomplete records.3**Select** the lowest-frequency, near-zero-stress loops for quasi-static J–A calibration.4Define *θ*_JA_ = [*k*, *c*, *a*, *α*]^T^ ∈ *Ω*_JA_.5**For each** Bayesian candidate *θ*_JA_ **do**: simulate the quasi-static J–A response and evaluate *J*_QS_(*θ*_JA_).6**End for**; set $\theta_{\mathrm{JA}}^{*}={\mathrm{argmin}}_{\theta \in \Omega_{\mathrm{JA}}}J_{\mathrm{QS}}\left(\theta \right).$7Keep *σ*_0_ fixed as a material property; perform sensitivity analysis only when enabled.8**If** dynamic calibration is enabled, estimate the frequency-loss scales and update *K*_1_ and *K*_2_; **end if**.9**For each** experimental loop *i* = 1, …, *N_L_* **do**:10Simulate the calibrated J–A, J–A + Sablik, and—when required—dynamic J–A + Sablik responses.11Resample all trajectories on the same cycle grid; select $B_{i}^{\mathrm{phys}}\left(t\right)$ and calculate ${\dot{H}}_{i}\left(t\right)$, field direction *d_i_*(*t*), and phase *φ_i_*(*t*).12**End for**.13Perform a sample-aware grouped split into training, validation, and unseen test subsets.14Fit every normalization statistic **using the training subset only**.15**For each** training/validation loop **do**: set $\Delta B_{i}\left(t\right)=B_{i}^{\exp}\left(t\right)-B_{i}^{\mathrm{phys}}\left(t\right)$ and construct $x_{i}\left(t\right)={\left[H_{n},B_{\mathrm{phys},n},{\dot{H}}_{n},d,\log_{10}{\left(f\right)}_{n},\sigma_{n},p_{n},\sin\left(2\pi \varphi \right),\cos\left(2\pi \varphi \right)\right]}^{T}.$16**End for**.17Initialize the CRRN: Conv1D(64,7) → Conv1D(64,5) → BiLSTM(128) → BiLSTM(64) → FC(64) → linear residual output, with layer normalization and dropout.18**For** epoch = 1, …, *E*_max_ **do**: update the network with Adam on mini-batches; monitor validation loss and stop when the patience criterion is reached; **end for**.19**For each** unseen test loop *i* **do**: predict ${\hat{\Delta B}}_{i}\left(t\right)=N_{\psi^{*}}\left(x_{i}\left(t\right)\right)$, undo residual normalization, and constrain the correction.20Reconstruct $B_{i}^{\mathrm{hyb}}\left(t\right)=B_{i}^{\mathrm{phys}}\left(t\right)+{\hat{\Delta B}}_{i}\left(t\right)$ and enforce endpoint closure of the predicted cycle.21Compute MAE, RMSE, and *R*^2^ for the physics and hybrid predictions; store per-loop/grouped results and perform the paired test when applicable.22**End for**; **return** $\theta_{\mathrm{JA}}^{*}$, $N_{\psi^{*}}$, *B*^hyb^, and the final test metrics.

The physics-based model and residual-learning workflow are implemented in MATLAB. The experimental BH loops are used for the modelling study and partitioned into training, validation, and test sets using proportions of 70%, 15%, and 15%, respectively. The model uses a sequence length of 512 points. The training loops and test loops presented in this manuscript are the best-reconstructed examples from their respective sets, selected according to R^2^ for visual illustration. The main training settings are summarized in Table 4. This fixed split does not cover all possible generalization scenarios, as it does not systematically assess performance across different data partitions or under entirely unseen operating conditions. We adopted this approach to limit the computational cost of repeated model training associated with cross-validation and alternative splitting strategies. The primary scope of this paper is to evaluate the contribution of CNN–BiLSTM residual learning to improving the JA–Sablik model within the investigated experimental domain. Future work will extend this assessment through repeated cross-validation and dedicated tests on held-out stress levels, excitation frequencies, material orientations, and additional steel grades.

As described in Section 2, the loops used in this study are drawn from the full experimental database covering three material orientations: rolling direction (RD, 0°), diagonal direction (DD, 45°), and transverse direction (TD, 90°). The database includes excitation frequencies of 1, 50, and 100 Hz, nine mechanical stress levels from −20 to +20 MPa in 5 MPa increments, and multiple peak induction levels. The partition therefore draws on measurements collected under multiple magneto-mechanical conditions, rather than a single orientation or excitation frequency.

## 4. Results

This section presents the experimental and modelling results obtained with the proposed hybrid Jiles–Atherton–Sablik and CNN–BiLSTM framework. This section presents the results obtained with the hybrid Jiles–Atherton–Sablik and CNN–BiLSTM framework. It discusses the fixed material constants and calibrated model parameters, prediction accuracy on the test set, computational time, magnetostriction response, reconstructed hysteresis cycles, and training convergence. The calibrated Jiles–Atherton parameters are then discussed, followed by the predictive performance on unseen test data, the magnetostrictive response, and the reconstructed hysteresis cycles.

### 4.1. Physical and Optimized Model Parameters

Table 5 summarizes the fixed material and model constants used in the Jiles–Atherton–Sablik formulation. The saturation magnetization M_s_ and saturation magnetostriction λ_s_ are treated as fixed material quantities. The magnetostrictive formulation additionally uses the elastic constants c_11_ and c_12_, the crystallographic magnetostriction coefficients λ_100_ and λ_111_, and the hysteretic coefficients α′ and α″. The frequency-dependent terms are denoted K_1_ and K_2_ in the analytical formulation; the MATLAB implementation stores the corresponding dynamic coefficients as D_1_ and D_2_.

Table 6 presents the starting and optimized values of the four Jiles–Atherton parameters obtained through Bayesian calibration. The optimization modifies the parameter set jointly so that the simulated hysteresis trajectories better reproduce the experimental loops. Because k, c, a and α are coupled through the nonlinear hysteresis equations, their individual variations should be interpreted primarily as the result of a global calibration rather than as independent changes in material properties.

### 4.2. Performance and Comparison 

Table 7 compares the standalone Jiles–Atherton–Sablik physics model with the proposed hybrid model on unseen test data. The physics-based model reproduces the main hysteretic behaviour but retains noticeable trajectory errors under the combined effects of stress and frequency. Information on the error metrics and their interpretation is given in [30,31,32]. The hybrid model substantially improves the agreement with the experimental loops. Both pointwise error measures decrease, while the coefficient of determination increases markedly. The lower dispersion of the hybrid error also indicates more consistent predictive behaviour across the unseen test conditions. These results show that the residual CNN–BiLSTM corrects systematic discrepancies that remain after calibration of the analytical physics model. This improvement in accuracy comes with additional computational cost. On CPU, the standalone JA–Sablik calculation requires an average of 19.378 ± 2.661 ms per B–H cycle, whereas the CNN–BiLSTM residual correction requires an additional 92.026 ± 3.963 ms per cycle. The measured component times sum to approximately 111.404 ms per cycle, excluding feature preparation and final hybrid postprocessing. Thus, the hybrid approach offers improved prediction accuracy at a higher computational cost than the standalone model. These measurements provide an initial basis for assessing computational feasibility, while practical integration into finite element simulations requires evaluation of the complete prediction workflow.

Table 8 summarizes the relative improvement achieved by residual learning. The reductions in MAE and RMSE, together with the increase in R^2^, confirm that the hybrid architecture improves both the magnitude and overall fidelity of the reconstructed hysteresis response. The improvement is therefore not limited to a simple amplitude correction but reflects a better reproduction of the measured trajectory.

### 4.3. Magnetostriction Behaviour

The magnetostriction response is evaluated using the Sablik magneto-mechanical formulation. The predicted response evolves smoothly over the magnetic cycle, with distinct and continuous loading and unloading branches. At high magnetic fields, the response remains bounded and tends toward saturation. These features provide a qualitative assessment of the behaviour represented by the coupled model. Table 9 summarizes the observations; this assessment does not constitute quantitative validation against experimental magnetostriction measurements.

### 4.4. Reconstructed Hysteresis Cycles

Table 10 presents the three best-reconstructed hysteresis cycles from each of the training and test sets, ranked separately by R^2^. These loops were selected for illustration. The selected training cases show close agreement between predictions and experimental measurements, while the selected test cases demonstrate accurate reconstruction of loops excluded from network training. Among the displayed test cases, the loop with the highest peak induction has a lower R^2^, although these selected examples alone do not establish a generalized relationship between induction level and prediction accuracy.

### 4.5. Training Convergence

Table 11 summarizes the training convergence of CNN-BiLSTM residual network. The training loss decreases from an initial value of 0.3548 to a final value of 0.0036, with a best value of 0.0034, corresponding to a reduction of 99.05%. The validation loss decreases from 0.2386 to 0.0048, with a best value of 0.0039, corresponding to a reduction of 98.36%. The close agreement between the final training and validation losses (0.0036 vs. 0.0048) indicates that the model achieves good generalization without significant overfitting. The training process converges after 63 epochs, demonstrating the efficiency of the Adam optimizer and the effectiveness of the early stopping strategy.

### 4.6. Visual Comparison of Hysteresis Cycles

**Figure 9:**
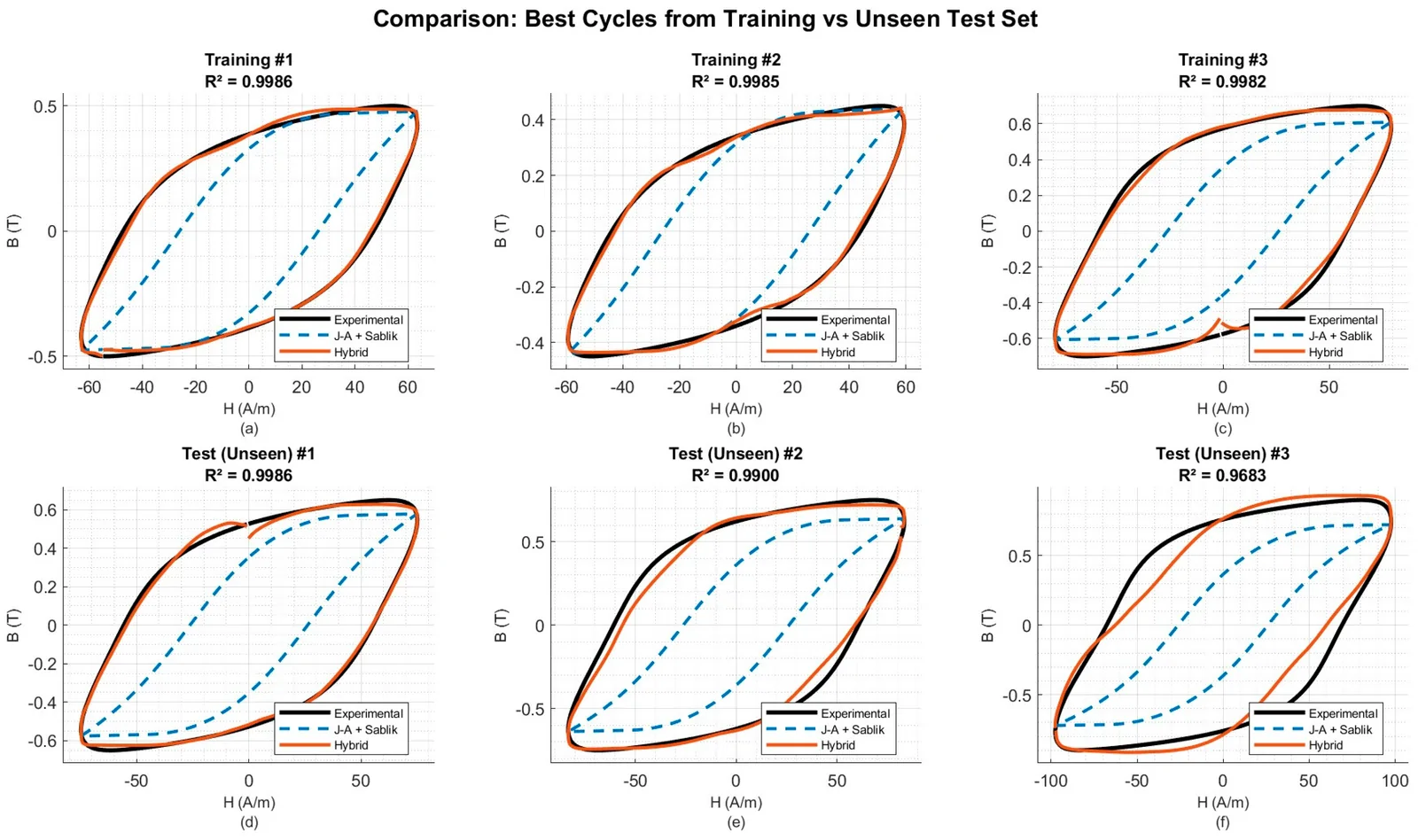
Comparison of representative hysteresis cycles from the training and unseen test sets: (**a**) DD, 100 Hz, 500 mT (R^2^ = 0.9986); (**b**) DD, 100 Hz, 450 mT (R^2^ = 0.9985); (**c**) DD, 100 Hz, 700 mT (R^2^ = 0.9982); (**d**) DD, 100 Hz, 650 mT (R^2^ = 0.9986); (**e**) DD, 100 Hz, 750 mT (R^2^ = 0.9900); and (**f**) DD, 100 Hz, 900 mT (R^2^ = 0.9683).

Figure 9 compares representative hysteresis cycles from the training and test sets using the experimental data, the JA–Sablik physical model, and the proposed hybrid model. For the training cases, the hybrid prediction follows the experimental loops very closely, showing that the residual correction effectively compensates for the discrepancies of the physical model. A similarly strong agreement is observed for the test cases, where the R^2^ values range from 0.9683 to 0.9986. These results indicate that the hybrid model not only reproduces the training data accurately but also preserves a high level of predictive performance on unseen conditions, demonstrating its ability to improve the JA–Sablik baseline while maintaining good generalization.

### 4.7. Training and Validation Loss Convergence

**Figure 10:**
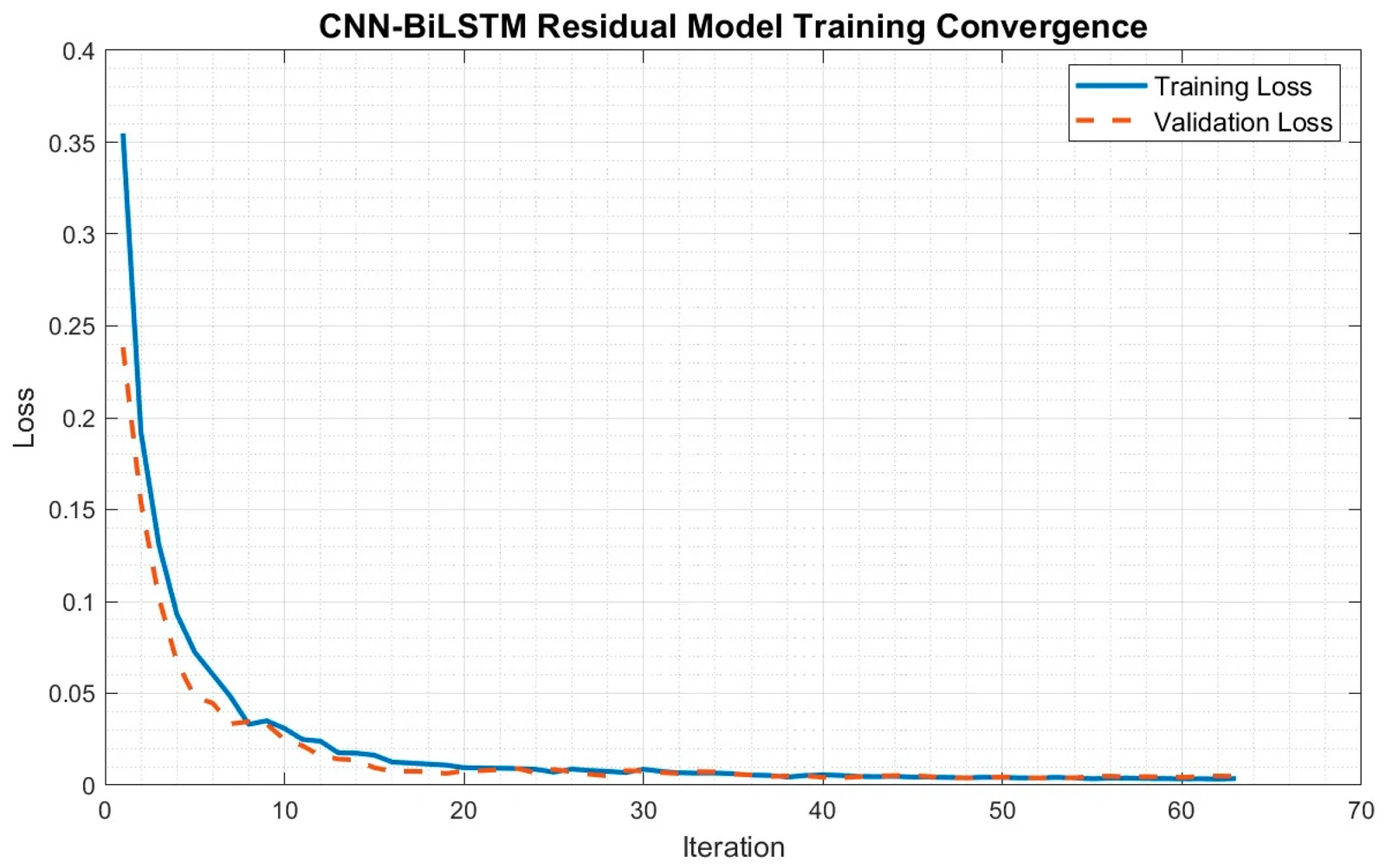
Loss function convergence during training and validation.

Figure 10 illustrates the convergence of the training and validation losses during CNN-BiLSTM optimization. Both losses decrease rapidly during the early stages of training and then decline more gradually as the model approaches convergence. The training loss decreases from approximately 0.35 to below 0.01 within the first 20 epochs, while the validation loss follows a comparable trend. Training is stopped at epoch 63 when no further improvement in the validation loss is observed. At this stage, the final training and validation losses are 0.0036 and 0.0048, respectively. The small gap between the two curves suggests that the model maintains good generalization throughout training, with no clear indication of substantial overfitting. This behavior also supports the effectiveness of the adopted regularization and early-stopping strategy.

**Figure 11:**
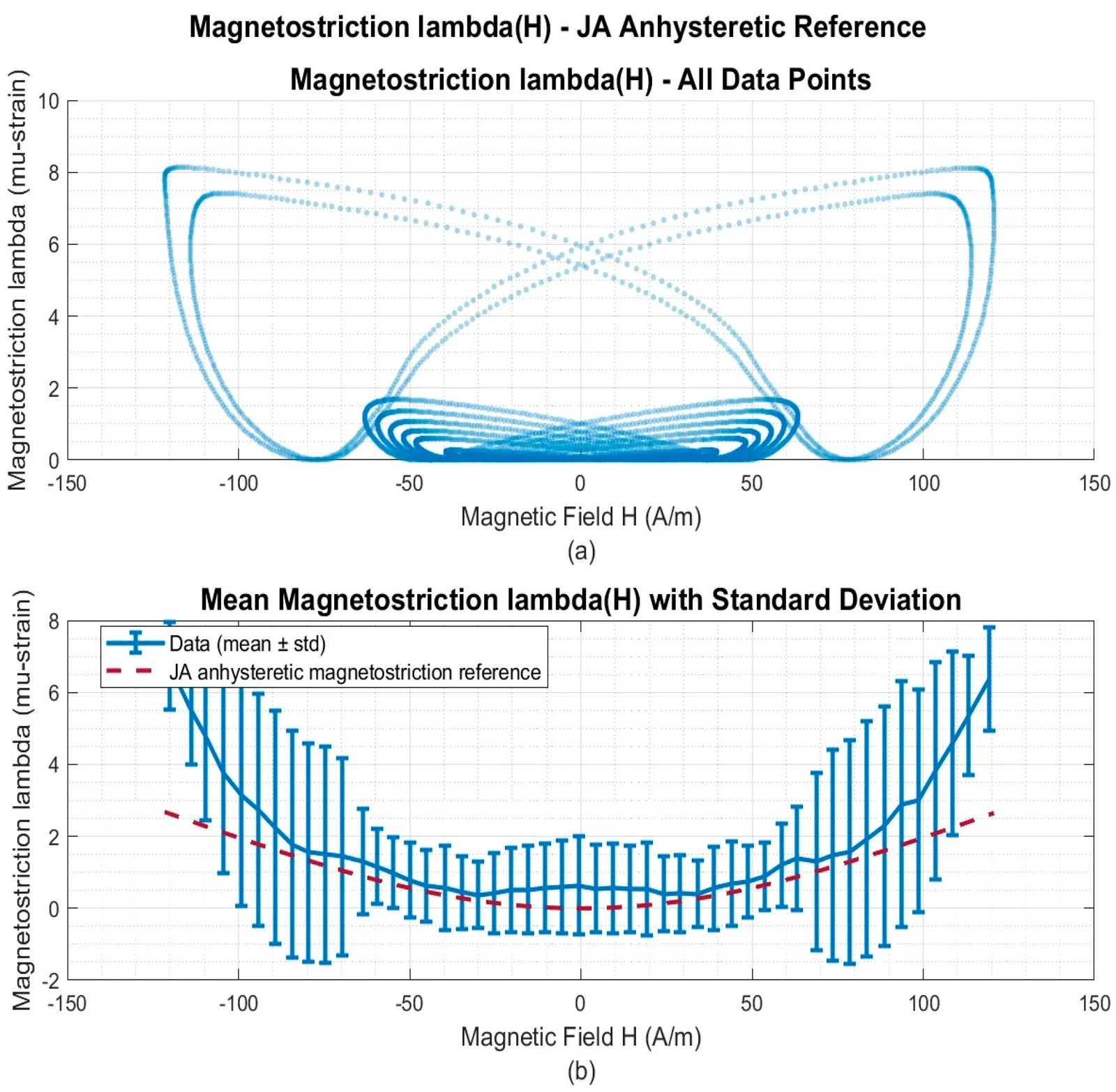
Magnetostriction lambda (H)-JA anhysteric reference: (**a**) Magnetostriction (H) of all data points; (**b**) Mean magnetostriction *λ_s_* (H) with standard deviation.

Figure 11 illustrates the magnetostriction response calculated using the Sablik formulation. Figure 11a shows the magnetostriction values over the hysteresis cycle, revealing the characteristic butterfly-shaped behavior commonly associated with ferromagnetic materials. The calculated values range from approximately 3 × 10^−15^ to 8 × 10^−6^. Figure 11b presents the mean magnetostriction together with its standard deviation, showing a gradual increase with magnetic field followed by a tendency toward saturation at higher field levels. The JA anhysteretic reference follows the same overall trend, supporting the physical consistency of the adopted Sablik magneto-mechanical formulation. The observed dispersion reflects the variation in magnetostriction response across the different operating conditions considered in the dataset.

**Figure 12:**
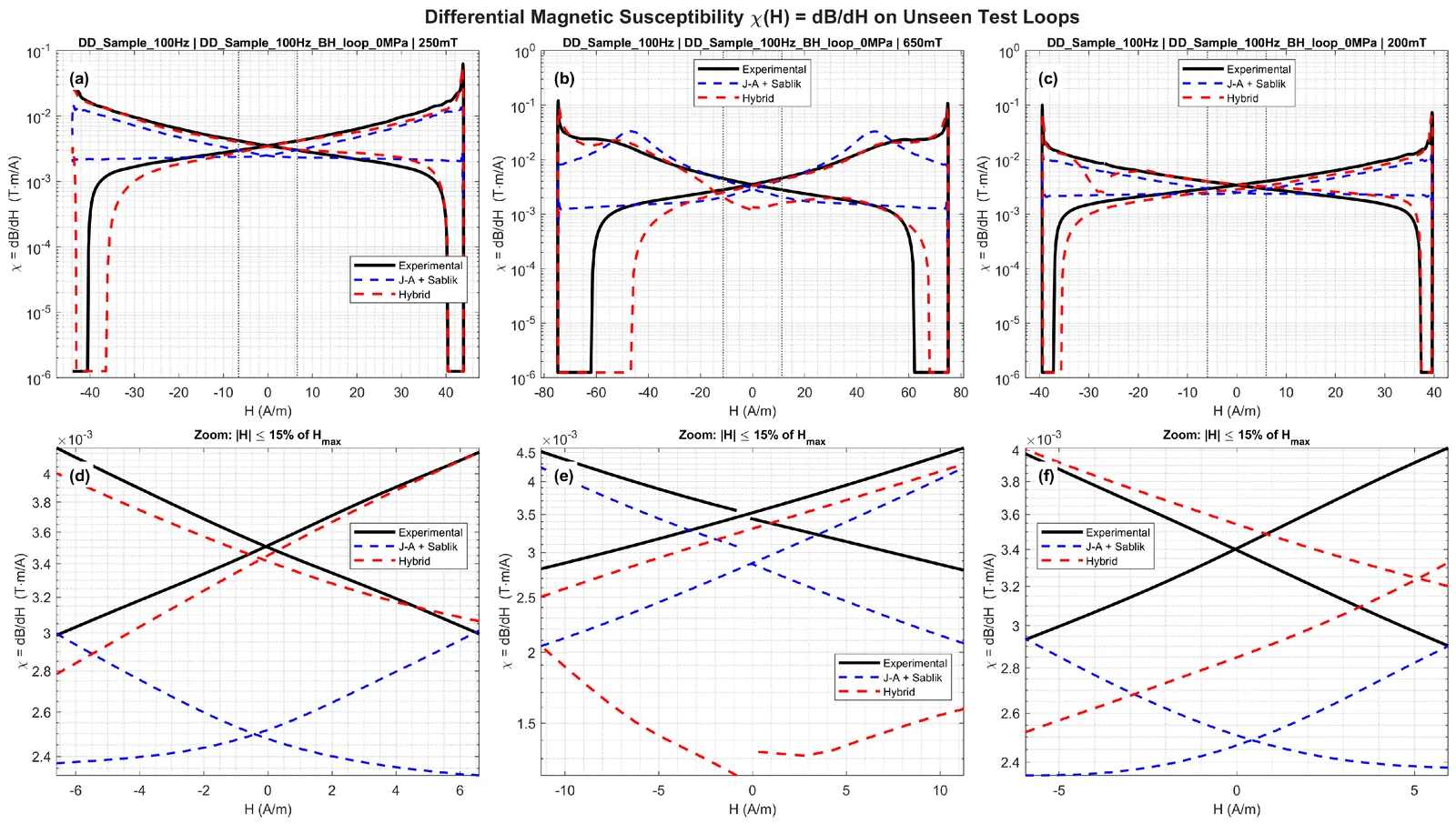
Differential magnetic susceptibility for representative unseen test loops: (**a**–**c**) comparison of the experimental results, the Jiles–Atherton–Sablik physics prediction, and the hybrid physics–CNN-BiLSTM prediction; (**d**–**f**) enlarged views of panels (**a**), (**b**), and (**c**), respectively. The enlarged panels highlight local variations in the magnetic response and allow a clearer assessment of the agreement between the predicted and experimental behaviours.

Traditional error metrics alone do not fully indicate whether the hybrid correction improves the actual shape of the predicted hysteresis loop. For this reason, the differential magnetic susceptibility is examined as an additional indicator of the local magnetic behaviour. Figure 12a–c compares the experimental response, the Jiles–Atherton–Sablik physics model, and the proposed hybrid prediction for representative unseen test cases. The results show that the physics-based model reproduces the general behaviour of the experimental curves, while some differences remain in the regions where the magnetic response changes rapidly. The hybrid model provides a noticeably closer agreement with the experimental curves, particularly around the characteristic peaks and transition regions. The corresponding zoomed views in Figure 12d–f further confirm that the correction introduced by the CNN-BiLSTM improves the local shape of the predicted response rather than simply modifying its overall amplitude. These results therefore provide additional evidence of the good predictive capability of the proposed hybrid model.

**Figure 13:**
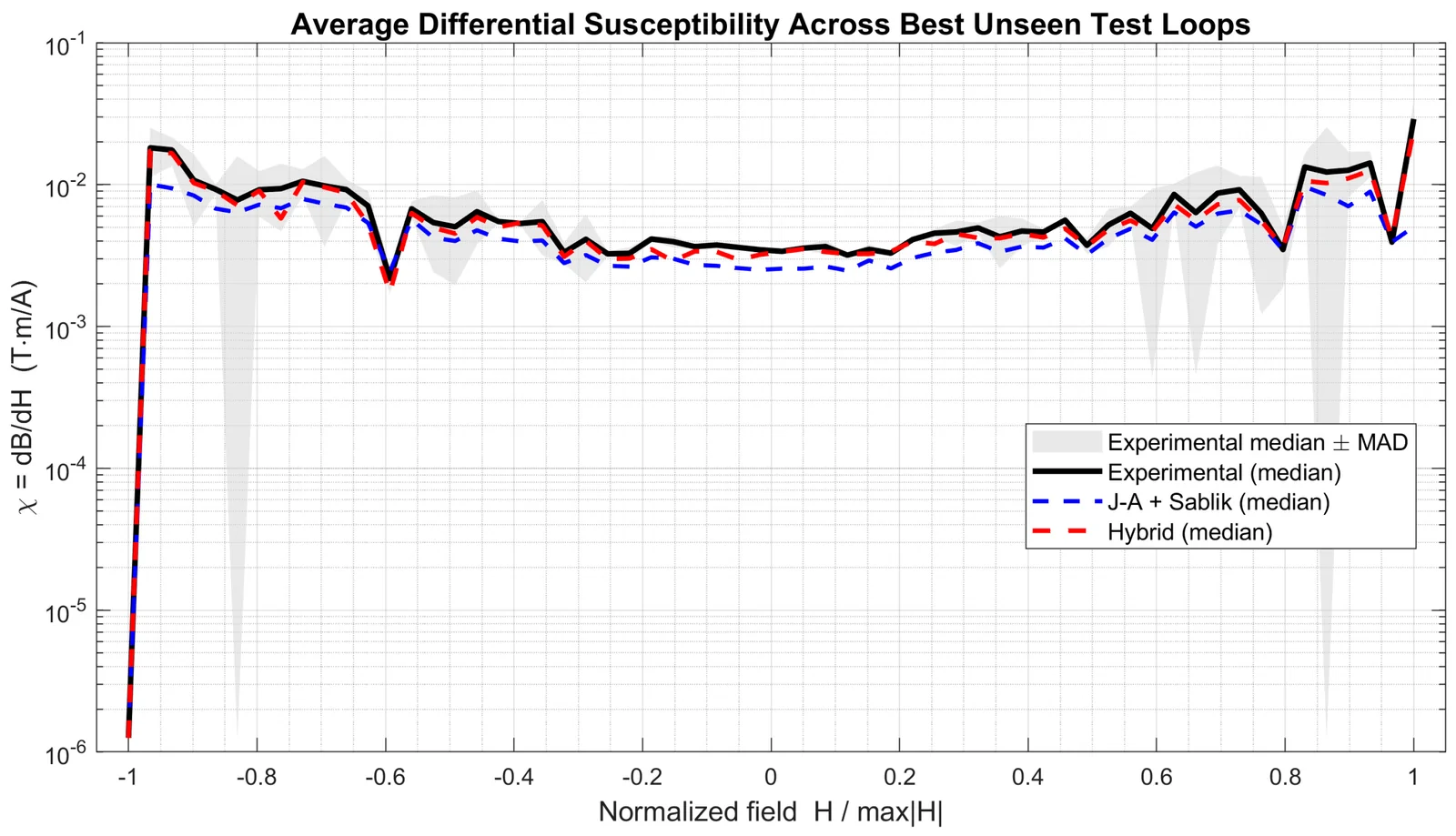
Aggregated differential magnetic susceptibility over the unseen test data. The figure compares the experimental response with the Jiles–Atherton–Sablik physics model and the proposed hybrid prediction, together with the experimental variability range.

To verify that the behaviour observed in the representative loops is maintained over the unseen test data, Figure 13 presents an aggregated comparison of the differential magnetic susceptibility. The experimental response is compared with the predictions obtained from the physics-based model and the proposed hybrid model. Overall, the hybrid prediction follows the experimental tendency more closely and remains within the range of variability observed in the experimental data over most of the investigated field range. In particular, the hybrid model better reproduces the characteristic changes in susceptibility and reduces the systematic deviations observed with the physics model alone. The agreement between the experimental and hybrid curves confirms that the residual network improves the magnetic response consistently across the test data rather than correcting only a limited number of individual loops. Taken together, Figure 12 and Figure 13 provide complementary evidence that the proposed hybrid approach produces more accurate and physically consistent results than the Jiles–Atherton–Sablik model used alone.

## 5. Comparison and Limitations

Building on the results presented above, this section places the proposed JA–Sablik/CNN–BiLSTM framework in the context of recent approaches that combine physical modelling and machine learning. It compares their modelling scope, treatment of magneto-mechanical effects, validation conditions, and reported performance. It then discusses the limitations of the study and directions for further validation and practical application.

### 5.1. Comparative Analysis

The proposed JA-Sablik/CNN-BiLSTM framework was compared with the four recent studies reviewed in Section 1.2 [7,8,9,10]. Because these studies do not address identical prediction targets, materials, operating ranges, or error definitions, their numerical metrics should not be interpreted as a strict benchmark on a common dataset. Nevertheless, the comparison is useful for evaluating modelling scope, physical content, learning strategy, validation breadth, generalization, and reported predictive accuracy. On this basis, the present work provides the most complete combination of stress-aware magnetic physics, dynamic excitation information, sample-orientation variability, and residual deep learning among the compared studies, while maintaining competitive accuracy on unseen B-H loops.

In terms of magnetic-loop accuracy, Li et al. [7] reported R2 values above 0.98 in most sinusoidal validation cases, with core-loss errors generally around 5%. Their hybrid JA-Preisach-BPNN model was also tested under distorted excitation; for a 50 Hz waveform containing a third harmonic, the reported R2 decreased to 0.9174 and the core-loss error reached −8.6%. The model required approximately 9 min of BPNN training per material and about 10–12 s per hysteresis cycle during computation. By comparison, the present JA-Sablik/CNN-BiLSTM model achieves an overall unseen-test R2 of 0.9856, MAE of 0.0458 T, and RMSE of 0.0616 T, while the best unseen cycle reaches R2 = 0.9986. More importantly, these results are obtained while explicitly accounting for mechanical stress, excitation frequency, and RD/DD/TD orientation effects, which are not included simultaneously in [7]. Thus, the principal advantage over [7] is not merely a similar high goodness-of-fit, but a broader magneto-mechanical operating domain and a residual-learning architecture that corrects a stress-aware physical baseline rather than coupling two hysteresis models.

Ni et al. [8] demonstrated that a JA-NARX architecture can preserve interpretability while accurately reproducing asymmetric and rate-dependent hysteresis. At 60 Hz, the reported peak-to-valley prediction error was 0.8190 micrometres, corresponding to only 0.82% of the full-scale displacement range; the mean error was 0.0737 micrometres and the error standard deviation was 0.1231 micrometres. In an additional model comparison, JA-NARX required 18 s, compared with 432 s for Bouc-Wen and 587 s for the conventional JA implementation. These results demonstrate strong efficiency and dynamic modelling capability. However, ref. [8] concerns voltage-displacement hysteresis of piezoelectric actuators rather than magnetic B-H hysteresis. Consequently, it does not model magnetostriction, stress-induced changes in magnetic domains, electrical-steel anisotropy, or the coupling between applied stress and magnetic excitation. The present work extends the physics-AI concept to magneto-mechanical hysteresis prediction and directly predicts BH loops under combined magnetic and mechanical conditions.

Hong et al. [9] provide the closest conceptual precedent for residual physics-informed learning under mechanical loading. Their model derives a physical magnetostriction equation from the JA formulation and uses a PINN to learn the residual of the governing physical equation. For a three-hidden-layer network with 30 neurons and a balanced physics/data weight, the normwise relative errors reported for 1.1 UN, UN, and 0.9 UN were approximately 0.11, 0.17, and 0.39, respectively; the corresponding ANN errors were 0.17, 0.34, and 0.76. The PINN therefore improved generalization when voltage conditions differed from the training data. However, the authors also state that the method is particularly suitable when stress remains constant and voltage changes only slightly. The study focuses on magnetostriction of grain-oriented steel under tensile stress and does not predict complete BH loops under tensile, compressive, and zero-stress states. In contrast, the present framework learns the residual directly in magnetic induction and is validated on non-oriented steel under multiple stresses, three sample orientations, and multiple frequencies, giving it broader applicability for BH hysteresis prediction.

She et al. [10] address a different but complementary problem: rapid inverse identification of JA parameters. Their physics-guided KAN-EfficientNet model reported parameter-inversion errors below 2.41% and an inference time of approximately 0.3 s, compared with about 35 s for the theoretical inverse JA procedure. The model was evaluated using a held-out test set of 2304 samples and experimentally on three T22 steel specimens. The proposed network contained approximately 3.91 million parameters, required about 529.58 MMac, and had a reported training time of 38,230 s. These results establish a strong advantage for fast parameter inversion. However, ref. [10] does not directly solve the forward prediction problem of stress- and frequency-dependent B-H hysteresis, and its physical guidance is used mainly to constrain inversion of k, c, *a*, and alpha. The present work instead optimizes the JA parameters once, retains Sablik quantities as material-based physical parameters, and then uses CNN-BiLSTM residual learning to correct the forward JA-Sablik prediction. The two strategies are therefore complementary, but the present approach addresses a broader prediction task involving magneto-mechanical response rather than parameter identification alone.

The quantitative results of the present model further support the value of this architecture. Relative to its own standalone JA-Sablik baseline, the hybrid correction reduces MAE from 0.2722 T to 0.0458 T (83.19% reduction) and RMSE from 0.3349 T to 0.0616 T (81.61% reduction), while R2 increases from 0.6273 to 0.9856. The standard deviation of MAE is also reduced from 0.0662 T to 0.0350 T. This within-dataset comparison is more rigorous than cross-paper comparison because both models are evaluated on the same unseen samples. It confirms that the CNN-BiLSTM stage is not merely fitting the training data but is learning systematic residual structure left by the physics model. Training and validation losses decrease by 99.05% and 98.36%, respectively, and convergence occurs after 63 epochs, with no strong divergence between the two curves.

The evidence in Table 12 supports the conclusion that the present work provides the main superiority of the capabilities considered here. Reference [7] provides strong dynamic magnetic accuracy but excludes mechanical stress; Reference [8] combines JA structure and recurrent learning but for a non-magnetic piezoelectric system; Reference [9] introduces stress-aware residual physics learning but predicts magnetostriction rather than complete B-H hysteresis and is mainly validated for tensile stress with limited voltage variation; and [10] provides very fast, accurate JA parameter inversion but does not perform forward magneto-mechanical residual correction. The present model is the only one among these five approaches that simultaneously combines a JA-Sablik stress-aware magnetic baseline, dynamic/frequency information, RD/DD/TD orientation variability, tensile and compressive loading, grouped unseen-data validation, and CNN-BiLSTM residual learning. Its R2 of 0.9856 on the unseen test set and best-cycle R2 of 0.9986 are competitive with the highest loop-fitting values reported in [7], while the 83.19% reduction in MAE relative to the same physics baseline provides direct evidence that residual learning materially improves the model under identical test conditions. Therefore, the main superiority of the proposed framework lies in the combination of physical completeness, experimental coverage, and unseen-data prediction accuracy rather than in claiming universal numerical dominance over studies that solve different tasks.

### 5.2. Limitations of the Present Study

Despite the favourable comparison, several limitations should be stated explicitly.

1. The present validation is based on one non-oriented electrical-steel dataset. Although the test files are unseen during training and the model achieves high R2 values, broader external validation on additional steel grades, manufacturers, thicknesses, and magnetic textures is required before claiming material-independent generalization.
2. The investigated frequency range is limited to the experimental conditions available in the dataset (1, 50, and 100 Hz), and the stress states are bounded by the tensile/compressive levels used during measurement. The CNN-BiLSTM residual correction may therefore extrapolate poorly beyond the ranges represented during training. This is a general limitation of residual-learning models: the physical baseline can remain meaningful outside the training domain, whereas the learned residual has no guarantee of preserving physical behaviour under severe extrapolation.
3. The standalone JA-Sablik baseline still exhibits a relatively large MAE of 0.2722 T and R^2^ of 0.6273. The hybrid model corrects this deficiency very effectively, but the magnitude of the improvement also indicates that a substantial portion of the final accuracy is supplied by the neural residual. Consequently, the final prediction is less directly interpretable than the physical component alone. Future work should investigate physics-constrained residual regularization, uncertainty quantification, and explicit monotonicity/symmetry constraints to preserve more physical structure in the learned correction.
4. The reported component timings provide an initial estimate of computational cost. Further assessment should measure the complete prediction workflow, including feature preparation and postprocessing, and evaluate its integration into finite-element simulations.

**Table 12:** Detailed comparison of the proposed model with recent physics-AI hysteresis approaches.

Study	Target/Material	Physical Basis	Learning Strategy	Stress/Magnetomechanics	Dynamic/Frequency	Validation/Data	Key Reported Metrics	Main Limitation
Li et al. [7]	Dynamic magnetic hysteresis; GO SiFe, NO SiFe, amorphous ribbon	Dynamic JA + Preisach + eddy/excess loss terms	BPNN learns dynamic proportion between JA and Preisach contributions	No external mechanical-stress model; no Sablik coupling	50 Hz sinusoidal; DC bias and third-harmonic distorted excitation	100 loops/material; 80% training, 20% verification; 3 materials	Most sinusoidal cases: R2 > 0.98, core-loss error ~5%; distorted harmonic case: R2 = 0.9174, loss error = −8.6%; ~9 min training/material; ~10–12 s/cycle	Strong dynamic modelling but no combined mechanical stress/orientation treatment; BPNN couples two models rather than learning residual error
Ni et al. [8]	Rate-dependent hysteresis of piezoelectric positioning actuator	JA transformed into JANN structure	JANN + NARX hybrid neural network	Not magnetic stress coupling; no magnetostriction model	3, 15, 30, 60 Hz excitation	70/15/15 train/validation/test split on experimental piezoelectric data	At 60 Hz: PV error = 0.8190 um (~0.82% full scale), AVG = 0.0737 um, STD = 0.1231 um; comparative runtime 18 s	Excellent explainability and dynamics, but system is piezoelectric rather than stressed electrical steel; selected NARX settings acknowledged as sub-optimal
Hong et al. [9]	Magnetostriction of GO electrical steel	JA-derived physical magnetostriction model with elastic energy	PINN learns residual of physical equation	Tensile stress included (not compression); stress strongly influences magnetostriction	Excitation-voltage variation; magnetostriction frequency effects	Training at 20 MPa and 1.1 UN; 1000 points; 5000 epochs; tests at UN and 0.9 UN	PINN relative error (3 × 30, wL = 1): 0.11 at 1.1 UN, 0.17 at UN, 0.39 at 0.9 UN; ANN: 0.17, 0.34, 0.76	Predicts magnetostriction, not full B-H loops; best suited to constant stress and small voltage changes; GO steel only
She et al. [10]	Inverse identification of JA parameters; ferromagnetic/T22 steel	Inverse JA equations embedded as physics constraints	Physics-guided KAN-EfficientNet	No explicit Sablik stress coupling in forward B-H prediction	Designed for rapid parameter inversion; not a dynamic forward hysteresis model	Held-out 2304-sample test set plus 3 experimental T22 specimens	Parameter-inversion error <2.41%; inference ~0.3 s vs. ~35 s theoretical inversion; 3.91 M parameters; 529.58 MMac; training 38,230 s	Targets parameter inversion rather than direct stress/frequency-dependent hysteresis prediction; large initial DL training cost
Present work	B-H hysteresis of non-oriented electrical steel; RD, DD, TD	JA-Sablik magneto-mechanical baseline + dynamic loss terms	CNN-BiLSTM learns residual B error while preserving physical baseline	Tension, compression, and 0 MPa explicitly included; Sablik magnetostriction/stress coupling	1, 50, and 100 Hz; frequency included in residual features	Grouped 70/15/15 split; unseen test loops; multiple orientations/stresses/frequencies	Unseen test: MAE = 0.0458 T, RMSE = 0.0616 T, R2 = 0.9856; best unseen R2 = 0.9986; MAE reduction vs. physics = 83.19%	Broader combined coverage and strong unseen-loop accuracy, but currently limited to one steel dataset/material family and a relatively small held-out test set; external cross-material validation remains needed

Note: The numerical metrics are not directly interchangeable because the studies predict different physical quantities, use different units, datasets, materials, and validation protocols. The table therefore supports a comparison of accuracy together with modelling scope, physical completeness, and generalization rather than a single scalar ranking.

## 6. Conclusions

This paper has presented a hybrid modelling framework for magneto-mechanical hysteresis prediction in non-oriented electrical steel, combining a Jiles–Atherton–Sablik (JA-Sablik) physical model with a deep residual correction based on a CRRN. The proposed architecture preserves the physical interpretability of the analytical hysteresis model while using deep learning exclusively to correct the remaining discrepancy between the physics-based prediction and experimental measurements. The framework was validated using a comprehensive experimental database comprising non-oriented electrical steel sheets tested under three orientations (RD, DD, TD), nine mechanical stress levels (−20 MPa to +20 MPa), three excitation frequencies (1 Hz, 50 Hz, 100 Hz), and multiple peak induction levels, totaling over 2000 BH loop files. The four Jiles–Atherton parameters (k, c, *a*, α) were optimized using Bayesian optimization while Sablik physical parameters were maintained as material-based fixed properties. The hybrid model achieved a Mean Absolute Error (MAE) of 0.0458 T, a Root Mean Square Error (RMSE) of 0.0616 T, and a coefficient of determination (R^2^) of 0.9856 on unseen test data, representing improvements of 83.19% in MAE, 81.61% in RMSE, and 57.13% in R^2^ compared to the standalone JA-Sablik model. The best reconstructed cycle on unseen data reached R^2^ = 0.9986, showing accurate reconstruction of the selected test cycle. The training process converged after 63 epochs with loss reductions exceeding 98%, indicating effective learning without overfitting. The framework combines stress-aware physical modelling with sequential residual learning for BH loop prediction. Future work should focus on external validation on additional steel grades, physics-constrained regularization, deployment for finite-element simulations, and extension to rotational magnetization and minor loop prediction.

## Figures and Tables

**Table 1:** Summary of representative recent studies and identified limitations.

Reference	Main Contribution	Main Advantage	Remaining Limitation Relative to This Study
Li et al. [7]	Hybrid dynamic JA-Preisach model coupled by a BPNN; represents domain-wall motion, domain rotation and eddy-current effects and is validated under sinusoidal and distorted excitations.	Combines complementary physical mechanisms with neural coupling and improves dynamic hysteresis/core-loss reproduction.	Does not include mechanical stress or Sablik type magneto-mechanical coupling; neural learning is used to couple models rather than correct a stress-aware physical residual.
Ni et al. [8]	Transforms the JA model into an interpretable JANN and combines it with NARX to capture asymmetric and rate-dependent hysteresis.	Retains physical meaning of JA-related network parameters while achieving accurate modelling with relatively low computational cost.	Validated for piezoelectric actuator hysteresis, not B-H hysteresis of stressed electrical steel; no magnetostriction or stress coupling.
Hong et al. [9]	Two-step physics-informed magnetostriction model: JA-based physical equation with elastic potential energy followed by PINN residual learning under tensile stress.	Embeds physical constraints and improves generalization over a conventional ANN, especially for unseen samples/voltage variations.	Targets magnetostriction of grain-oriented steel rather than complete B-H loop prediction; does not cover combined tension/compression, frequency and RD/DD/TD orientation effects.
She et al. [10]	Physics-guided KAN-EfficientNet for rapid inverse identification of JA parameters using inverse-model constraints.	Accelerates parameter inversion and improves accuracy/physical consistency compared with conventional data-driven or iterative approaches.	Focuses on parameter estimation rather than forward magneto-mechanical hysteresis prediction; no JA-Sablik residual correction across stress/frequency/orientation conditions.
This work	JA-Sablik physical baseline combined with CNN-BiLSTM residual learning; JA parameter optimization and validation on non-oriented electrical steel across orientation, stress and frequency conditions. (quasi-static and dynamic cases)	Preserves a physically interpretable magneto-mechanical baseline while using deep sequential learning only to correct the remaining modelling error.	Designed to address the combined gap identified above; transferability to other steel grades and operating ranges remains a topic for future validation.

**Table 2:** Geometric and physical properties of the evaluated non-oriented electrical steel samples.

Sample	Orientation	Length (mm)	Width (mm)	Thickness (mm)	Mass (g)	Density (kg/m^3^)	Cross Section Area (m^2^)
RD	0°	300.00	97.25	0.53	122.07	7845	5.1867 × 10^−5^
DD	45°	295.50	98.83	0.51	123.00	8135	5.1144 × 10^−5^
TD	90°	300.00	98.99	0.52	123.25	8046	5.0401 × 10^−5^

**Table 3:** Maximum peak induction values achieved.

Stress	1 Hz RD	1 Hz DD	1 Hz TD	50 Hz RD	50 Hz DD	50 Hz TD	100 Hz RD	100 Hz DD	100 Hz TD
0 MPa	1.50	1.45	1.50	1.30	1.30	1.35	1.20	1.10	1.15
−5 MPa	1.50	1.45	1.50	1.30	1.25	1.25	1.20	1.10	1.10
−10 MPa	1.50	1.45	1.45	1.30	1.25	1.25	1.10	1.05	1.05
−15 MPa	1.50	1.40	1.45	1.30	1.30	1.20	1.05	0.95	1.00
−20 MPa	1.45	1.30	1.35	1.30	1.10	1.30	0.95	0.90	0.90
+5 MPa	1.50	1.45	1.50	1.20	1.25	1.30	1.20	1.10	1.15
+10 MPa	1.50	1.45	1.50	1.20	1.25	1.25	1.20	1.10	1.20
+15 MPa	1.50	1.45	1.50	1.30	1.30	1.25	1.25	1.15	1.20
+20 MPa	1.50	1.45	1.50	1.30	1.30	1.25	1.20	1.15	1.20

**Table 4:** Training configuration.

Parameter	Value
Optimizer	Adam
Initial learning rate	1 × 10^−3^
Learning-rate drop period	35 epochs
Learning-rate drop factor	0.35
Mini-batch size	16
Maximum epochs	120
Validation patience	15 epochs
Dropout rate	0.15
Sequence length	512

**Table 5:** Fixed material and model constants used in Jiles–Atherton–Sablik formulation.

Parameter	Symbol	Value	Unit	Role
Saturation magnetization	*M_s_*	1,137,970	A/m	Fixed experimental/material quantity
Saturation magnetostriction	*λ_s_*	9.17 × 10^−6^	–	Fixed magnetostrictive quantity
Elastic coefficient	*c*_11_	202	GPa	Sablik elastic constant
Elastic coefficient	*c*_12_	122	GPa	Sablik elastic constant
Crystallographic magnetostriction	*λ*_100_	23 × 10^−6^	–	Magnetostriction constant
Crystallographic magnetostriction	*λ*_111_	−4.5 × 10^−6^	–	Magnetostriction constant
Hysteretic coefficient	*α′*	7.5 × 10^6^	–	Magnetostriction-loop parameter
Hysteretic coefficient	*α″*	3.5 × 10^5^	–	Magnetostriction-loop parameter
Dynamic coefficient 1	*K*_1_ *(D*_1_ *in MATLAB)*	5.03 × 10^−6^	model-scaled	Dynamic/anomalous-loss term
Dynamic coefficient 2	*K*_2_ *(D*_2_ *in MATLAB)*	6.72 × 10^−3^	model-scaled	Classical eddy-current term

**Table 6:** Optimized Jiles–Atherton parameters.

Parameter	Initial Value	Optimized Value	Variation
*k (A/m)*	69.0	29.2	−57.7%
*c*	0.0360	0.0658	+82.8%
*a (A/m)*	77.0	80.3	+4.3%
*α*	1.865 × 10^−4^	1.362 × 10^−4^	−27.0%

**Table 7:** Performance comparison on unseen test data.

Model	MAE (T)	RMSE (T)	R^2^	Std MAE (T)	Mean Time ± SD (ms/B–H Cycle)	Median (ms/B–H Cycle)
JA–Sablik physics model	0.272171	0.334915	0.627260	0.066215	19.378 ± 2.661	18.976
Hybrid JA–Sablik + CNN–BiLSTM	0.045755	0.061591	0.985616	0.035049	92.026 ± 3.963	90.734

**Table 8:** Improvement obtained by hybrid residual learning.

Metric	Improvement
MAE reduction	83.19%
RMSE reduction	81.61%
R^2^ improvement	57.13%

**Table 9:** Qualitative assessment of the predicted magnetostriction response.

Assessment Aspect	Observed Behaviour
Cycle continuity	Smooth evolution without apparent discontinuities
Loading and unloading	Distinct and continuous hysteretic branches
High-field behaviour	Bounded response with a tendency toward saturation
Overall response	Qualitatively consistent with the behaviour represented by the coupled model

**Table 10:** Best reconstructed hysteresis cycles.

Set	Sample	Frequency	Peak Induction	R^2^
Training #1	DD	100 Hz	500 mT	0.9986
Training #2	DD	100 Hz	450 mT	0.9985
Training #3	DD	100 Hz	700 mT	0.9982
Test #1	DD	100 Hz	650 mT	0.9986
Test #2	DD	100 Hz	750 mT	0.9900
Test #3	DD	100 Hz	900 mT	0.9683

**Table 11:** CNN–BiLSTM training convergence.

Quantity	Initial	Final	Best	Reduction
Training loss	0.354786	0.003633	0.003361	99.05%
Validation loss	0.238590	0.004835	0.003919	98.36%
Epochs		63		

## Data Availability

This work uses publicly available datasets under open-access licenses, which are cited in this manuscript under Refs. [14,15]. The raw data supporting the conclusions of this article will be made available by the authors on request.
